# The glucose-lowering effects of α-glucosidase inhibitor require a bile acid signal in mice

**DOI:** 10.1007/s00125-020-05095-7

**Published:** 2020-02-08

**Authors:** Yixuan Qiu, Linyan Shen, Lihong Fu, Jie Yang, Canqi Cui, Tingting Li, Xuelin Li, Chenyang Fu, Xianfu Gao, Weiqing Wang, Guang Ning, Yanyun Gu

**Affiliations:** 1 Shanghai National Research Centre for Endocrine and Metabolic Diseases, 197 Ruijin 2nd Road, Shanghai, 200025 China; 2 Shanghai Institute for Endocrine and Metabolic Diseases, Ruijin Hospital, Shanghai Jiao Tong University School of Medicine, 197 Ruijin 2nd Road, Shanghai, 200025 China; 3 Department of Endocrinology, East Hospital, Tongji University School of Medicine, Shanghai, China; 4 Key Laboratory of Systems Biology, Institute of Biochemistry and Cell Biology, Shanghai Institutes for Biological Sciences, Chinese Academy of Sciences, Shanghai, China

**Keywords:** Bile acid, Diabetes mellitus, Farnesoid X receptor, α-Glucosidase inhibitor, Pancreatic islet

## Abstract

**Aims/hypothesis:**

Bile-acid (BA) signalling is crucial in metabolism homeostasis and has recently been found to mediate the therapeutic effects of glucose-lowering treatments, including α-glucosidase inhibitor (AGI). However, the underlying mechanisms are yet to be clarified. We hypothesised that BA signalling may be required for the glucose-lowering effects and metabolic benefits of AGI.

**Methods:**

Leptin receptor (*Lepr*)-knockout (KO) *db*/*db* mice and high-fat high-sucrose (HFHS)-fed *Fxr* (also known as *Nr1h4*)-KO mice were treated with AGI. Metabolic phenotypes and BA signalling in different compartments, including the liver, gut and endocrine pancreas, were evaluated. BA pool profiles were analysed by mass spectrometry. The islet transcription profile was assayed by RNA sequencing. The gut microbiome were assayed by 16S ribosomal RNA gene sequencing.

**Results:**

AGI lowered microbial BA levels in BA pools of different compartments in the body, and increased gut BA reabsorption in both *db*/*db* and HFHS-fed mouse models via altering the gut microbiome. The AGI-induced changes in BA signalling (including increased activation of farnesoid X receptor [FXR] in the liver and inhibition of FXR in the ileum) echoed the alterations in BA pool size and composition in different organs. In *Fxr*-KO mice, the glucose- and lipid-lowering effects of AGI were partially abrogated, possibly due to the *Fxr*-dependent effects of AGI on decelerating beta cell replication, alleviating insulin hypersecretion and improving hepatic lipid and glucose metabolism.

**Conclusions/interpretation:**

By regulating microbial BA metabolism, AGI elicited diverse changes in BA pool composition in different host compartments to orchestrate BA signalling in the whole body. The AGI-induced changes in BA signalling may be partly required for its glucose-lowering effects. Our study, hence, sheds light on the promising potential of regulating microbial BA and host FXR signalling for the treatment of type 2 diabetes.

**Data availability:**

Sequencing data are available from the BioProject Database (accession no. PRJNA600345; www.ncbi.nlm.nih.gov/bioproject/600345).

**Electronic supplementary material:**

The online version of this article (10.1007/s00125-020-05095-7) contains peer-reviewed but unedited supplementary material, which is available to authorised users.

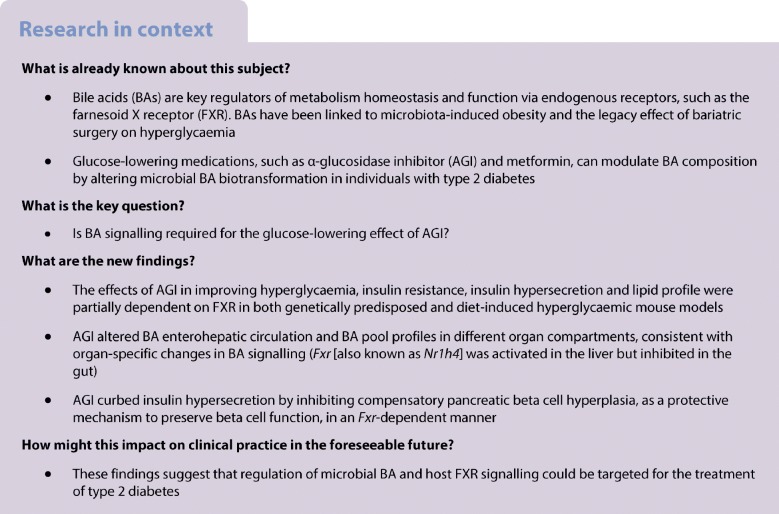



## Introduction

Medication and lifestyle management are effective remedies for type 2 diabetes, whereas there was no cure to treat type 2 diabetes in obese individuals until the legacy effect of bariatric surgery was discovered [1, 2]. Alterations in the gut microbiome and bile acids (BAs) are thought to mediate the metabolic benefits of surgery [3–5]. Notably, an oral glucose-lowering drug that is often prescribed in Asian populations, α-glucosidase inhibitor (AGI), known for lowering postprandial glucose excursions via inhibition of polysaccharide digestion [6, 7], has recently been shown to substantially affect the gut microbiota and intervene with microbial BA metabolism. These microbial changes have been associated with the glucose-lowering effects of AGI [8]. Similar changes have been found with metformin, a more widely used oral glucose-lowering drug [9, 10]. Therefore, as it is essential for the regulation of host BA pool composition, the role of gut microbiota BAs in mediating the therapeutic effects of oral glucose-lowering drugs or in managing type 2 diabetes awaits further clarification.

Via BA receptors, alterations in the BA pool regulate homeostasis of the host’s metabolism. As the key nuclear BA receptor, the farnesoid X receptor (FXR) is mainly expressed in the liver and gut [11–13], where it plays crucial roles in the regulation of BA biosynthesis, transport and reabsorption [14, 15]. Recent studies have recognised FXR as an important therapeutic target for treating metabolic diseases [3, 10, 16] and the efficacy of FXR-targeting drugs has been tested in individuals with non-alcoholic fatty liver disease (NAFLD) [17]. It has been confirmed that FXR is required for gut-microbiota-induced obesity in *Fxr*- (also known as *Nr1h4*) null germ-free mice [18]. On the other hand, the metabolic outcomes of targeting different BA receptors in different organs vary, owing to their pleotropic downstream effects and different affinities to various BA species [19]. Therefore, it is yet to be elucidated how interventions that are known for regulating bacterial BA metabolism (such as AGI) [8] could globally alter the host BA pool to merit metabolic outcomes.

As a key organ for maintaining glucose metabolism homeostasis, the pancreatic islet also expresses BA receptors to sense changes in the BA pool. Some ex vivo and in vitro studies have suggested a role for FXR in regulating insulin secretion [20, 21], but how dysfunctional pancreatic islets respond to AGI treatment in regard to BA signalling has not yet been tested. Hence, to address the above questions, we treated a genetically predisposed (leptin receptor-knockout [KO], *db*/*db*), as well as a nutrient-induced (high-fat high-sucrose [HFHS] diet) mouse model of hyperglycaemia with AGI. Further, with the aid of *Fxr*-KO mice, we investigated whether the glucose-lowering effect of AGI was mediated by its impact on BA pool composition and BA signalling, and how this altered liver lipid metabolism and gluconeogenesis, and pancreatic beta cell preserve.

## Methods

### Animal treatment and experimental procedures

Male C57BKS-*Lepr*^−/−^ (*db*/*db*) mice were purchased from the Model Animal Research Centre of Nanjing University (Nanjing Jiangsu, China). *Nr1h4*^*tm1Gonz/*^*J* (also known as *Fxr*-KO) mice were purchased from the Jackson Laboratory (RRID: IMSR_JAX:004144; Bar Harbor, ME, USA) and maintained on a C57BL/6J background. Only male animals were used in this study. Mice were maintained in a facility in Shanghai Jiao Tong University School of Medicine (Shanghai, China).

After purchase, 6-week-old *db/db* mice were maintained in the facility and subjected to chow diet (ReadyDietech, Shenzhen, China) for 2 weeks. Only mice that grew to have a bodyweight ≥25 g and a random blood glucose ≥15 mmol/l were included for further study. Eight-week-old *db*/*db* mice with similar bodyweights and random glucose levels were assigned to the AGI treatment group or the control group. For the AGI group, AGI (Acarbose; Huadong Medicine, Hangzhou, China) was mixed with the chow diet at a set concentration of 1000 mg/kg to make the daily dosage around 100 mg/kg bodyweight [22, 23]; mice were fed the AGI-mixed diet for 24 days. For the control group, vehicle treatment was provided for 24 days, which was the same chow diet as the AGI group but did not contain the AGI.

At 8 weeks of age, *Fxr*-KO mice and their wild-type littermates were fed an HFHS diet (ReadyDietech; 32% of kJ from fat, 25% of kJ from sucrose) for 10 weeks. See electronic supplementary material (ESM) Table 1 for details of diet formulas. After this, both genotypes were assigned to the AGI treatment or control group with HFHS diet, for another 4 weeks.

All mouse experiments were repeated three times in different litters, each time with 5–8 mice per group. Randomisation and blinding were not carried out for the animal experiments.

After treatment, all mice were euthanised using 10% (wt/vol.) chloral hydrate (10 μl/g body weight) after overnight (16 h) fasting. Blood samples were collected and the liver, ileum and pancreas of mice were harvested and either fixed in 4% (wt/vol.) paraformaldehyde or snap frozen and stored at −80°C.

Mice were fed ab libitum and housed in specific pathogen-free isolators at 23 ± 1°C with a 12 h light/dark switch. All animal protocols were approved by the Animal Care Committee of Shanghai Jiao Tong University School of Medicine.

### Blood glucose and insulin tolerance tests

After 6 h fasting (from 08:00 hours to 14:00 hours), 22-week-old *Fxr*-KO mice and their littermates were subjected to IPGTTs, in vivo glucose-induced insulin secretion (GIIS) tests (measured by ELISA) and ITTs [24]. See ESM Methods for more details.

### 16S ribosomal RNA gene sequencing of the gut microbiota and quantification of bacterial DNA

Bacterial DNA was extracted from colon content and faeces of mice as previously reported [25] and subjected to PCR amplification and Illumina MiSeq sequencing (San Diego, CA, USA). See ESM Methods for more details.

### Metabolomic study

BAs were extracted from plasma, cecum content, liver and ileum tissues of mice and analysed by ultra-performance liquid chromatography-tandem mass spectrometry (UPLC-MS/MS) using the Waters Acquity UPLC system (Waters, Milford, MA, USA) coupled to a Triple Quad 5500 tandem mass spectrometer (AB Sciex, Framingham, MA, USA), as described previously [8].

### Body composition

Body composition (fat and lean mass) of mice was assessed by an animal whole body composition analyser (EchoMRI 100H, Houston, TX, USA), according to the manufacturer’s instructions.

### Triacylglycerol and cholesterol measurement

Triacylglycerol and total cholesterol in both liver and plasma of mice were measured by the Quantification Colorimetric/Fluorometric Kit (Biovision, Milpitas, CA, USA), as previously reported [25]. See ESM Methods for further details.

### Oil Red O staining

Frozen mouse liver tissue sections with 5–10 μm thickness were subjected to Oil Red O and haematoxylin staining, using a standard protocol [26].

### Pancreatic beta cell mass measurement

Selected continuous pancreas sections from mice were immunolabelled with insulin and the area of staining in each section was analysed. See ESM Methods for further details.

### Pancreatic insulin content

Pancreatic insulin content was extracted from murine samples by acidic ethanol and assayed by a mouse insulin ELISA kit (Alpco, Salem, NH, USA). See ESM Methods for further details.

### Islet isolation and ex vivo GIIS assay

Mouse islets were isolated and handpicked to perform GIIS assays ex vivo [24]. After incubation with low glucose (3.3 mmol/l) or high glucose (16.7 mmol/l) for 1 h, the supernatant was collected or islets were lysed in acidic ethanol. The insulin level in supernatant and islet extracts were assayed by a mouse insulin ELISA kit (Alpco). See ESM Methods for further details.

### Immunolabelling assay

Immunolabelling of insulin, glucagon, MafA and urocortin-3 (UCN3) in mouse pancreas sections, and image processing were performed as previously described [24]. See ESM Methods for further details; antibody details are listed in ESM Table 2.

### Real-time quantitative RT-PCR

Real-time quantitative RT-PCR was used to determine the relative levels of gene transcription in murine samples. Expression levels were normalised to the housekeeping gene *36b4* (also known as *Rplp0*). See ESM Methods for further details. All primers used in this study were listed in ESM Table 3.

### Western blotting and antibodies

Tissues of mice were lysed, quantified, and blotted, as described previously [25]. See ESM Methods for further details; antibody details are listed in ESM Table 2.

### RNA Sequencing of pancreatic islets

The extracted RNA from isolated mouse primary islets was subjected to library construction and sequencing based on the BGISEQ-500 platform [27]. See ESM Methods for further details.

### Two-step hyperglycaemic clamp in humans with type 2 diabetes

The two-step hyperglycaemic clamp was employed to evaluate changes in beta cell function [28] in individuals with type 2 diabetes before (*n* = 10) and after (*n* = 9) AGI treatment. These individuals were enrolled in a 3 month intervention study to examine the glucose-lowering and gut microbiota effects of AGI (ClinicalTrials.gov. registration no. NCT01758471) [8]. Of the 106 participants in the original study, 20 individuals (*n* = 10 in the AGI group, *n* = 10 in the control group) additionally volunteered to take part in the clamp study; *n* = 19 completed the clamp study. All the study participants gave additional informed consent specifically for the clamp study. The trial conformed to the provisions of the Declaration of Helsinki and was approved by the ethics committees at each participating centre. See ESM Methods and ESM Table 6 for further details of the experimental procedures and participant characteristics.

### Antibiotic pre-treatment

A subset of *db*/*db* mice were subjected to an experiment comparing the effects of AGI treatment after antibiotic pre-treatment. Antibiotic pre-treatment of *db*/*db* mice was employed as a proxy for inducing germ-free status before AGI treatment. See ESM Methods for further details.

### Statistical analysis

Statistical calculations were conducted with SPSS v11.0 software (SPSS, Chicago, IL, USA). Two-group comparisons were analysed by two-tailed Student’s *t* test. Multiple groups and repeated measurements were analysed by single-tailed two-way ANOVA followed by Fisher’s least significant difference (LSD) post hoc test. Two-tailed Mann–Whitney *U* tests were used for analysing all BA metabolome data (including data from plasma, liver, ileum and cecum content samples of mice) and the gut microbiota data. All results are expressed as the mean±SEM. A *p* value of <0.05 was considered statistically significant.

## Results

### AGI induces significant alterations in the gut microbiome and BA pool in *db*/*db* mice

**Fig. 1:**
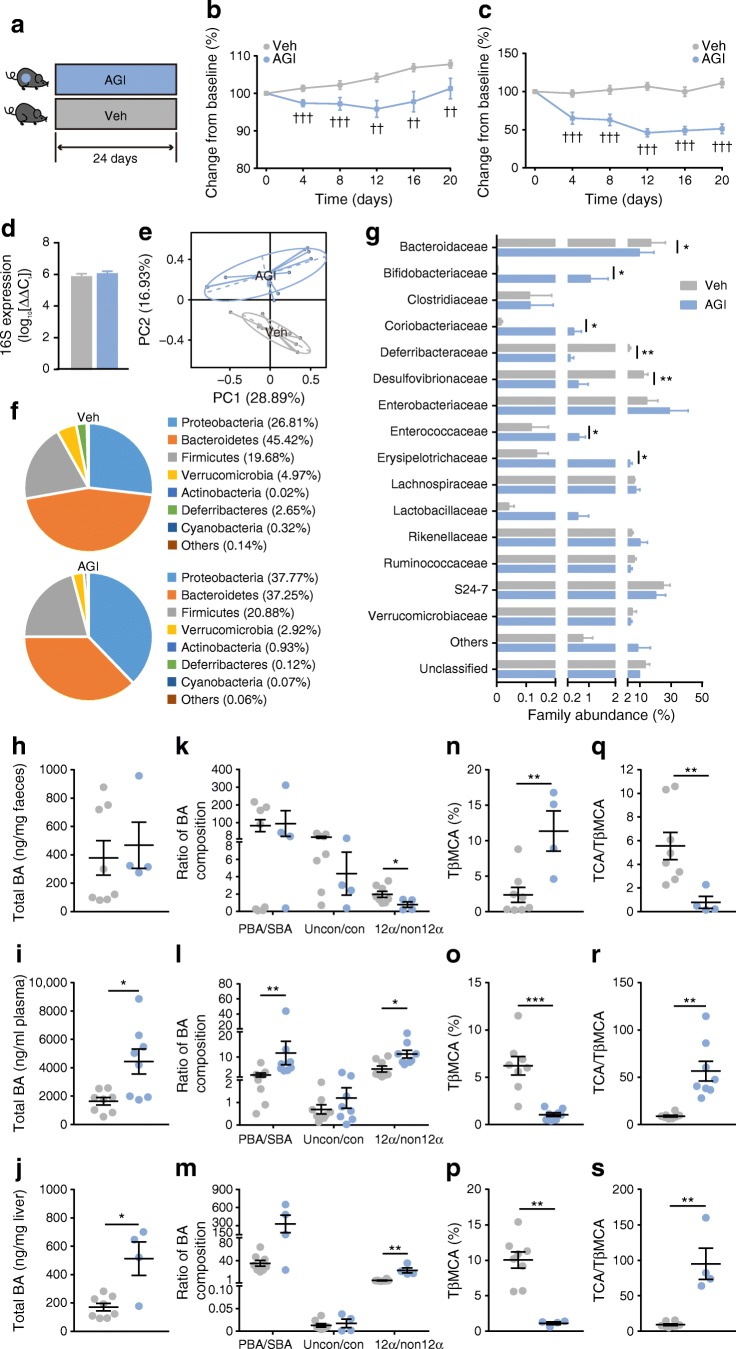
AGI altered gut microbiota and BAs and induced metabolic improvements in *db*/*db* mice. (**a**) Schematic of the study design. *db*/*db* mice were treated with vehicle (Veh) or AGI for 24 days. (**b**) Change in body weight from baseline. Veh, *n* = 19; AGI, *n* = 24. (**c**) Change in fed-state blood glucose from baseline. Veh, *n* = 19; AGI, *n* = 24. (**d**) Bacterial load in faeces samples, quantified by real-time quantitative RT-PCR amplifying the bacterial 16S ribosomal RNA gene. C_t_ values (shown on the *y*-axis) were normalised to a detection threshold of 30. Veh *n* = 9; AGI, *n* = 12. (**e**) Principal component analysis (PCA) based on operational taxonomic unit (OTU) composition. Each dot represents an individual mouse. Veh, *n* = 9; AGI, *n* = 12. (**f**) Pie plots representing gut microbiota composition at the phylum level in *db*/*db* mice after Veh or AGI treatment. Veh, *n* = 9; AGI, *n* = 12. (**g**) Gut microbiota composition at the family level. Veh, *n* = 9; AGI, *n* = 12. (**h**–**s**) BA content and composition in *db*/*db* mice in cecum content (**h**, **k**, **n** and **q**), plasma (**i**, **l**, **o** and **r**) and liver (**j**, **m**, **p** and **s**). Cecum: Veh, *n* = 8; AGI, *n* = 4. Plasma: *n* = 8 per group. Liver: Veh, *n* = 8; AGI, *n* = 4. Grey circles, Veh treatment; blue circles, AGI treatment. Data are presented as mean±SEM. ^††^*p* < 0.01, ^†††^*p* < 0.001, AGI vs Veh, two-way ANOVA; *p < 0.05, **p < 0.01, ***p < 0.001, AGI vs Veh, Mann–Whitney *U* test. 12α, 12α-hydroxylated BAs; con, conjugated BAs; non12α, non-12α-hydroxylated BAs; PC, principal component; uncon, unconjugated BAs

First, we treated *db*/*db* mice with AGI for 24 days (Fig. 1a). AGI consistently reduced body weight and blood glucose in *db*/*db* mice vs untreated controls (Fig. 1b, c) during the intervention, similar to its effects in humans [8, 29]. Body composition and plasma and liver lipids were all improved by the end of treatment, except for total cholesterol in the liver (ESM Fig. 1). With the same bacterial load (Fig. 1d), 16S ribosomal RNA sequencing revealed significant differences in gut microbiota composition between AGI treated and untreated mice (Fig. 1e), such as enriched phylum Actinobacterium and Proteobacterium and depleted phylum Bacteroides (Fig. 1f). Though not exactly the same, AGI-induced changes in relative abundance of Bacteroides and Bifidobacteriaceae in the *db*/*db* gut microbiome were similar to previous reports in human participants (Fig. 1g) [8]. The BA pool sizes were enlarged in the liver and plasma of *db*/*db* mice treated with AGI vs untreated controls, but these were not significantly different in cecum content (Fig. 1h–j). The enhanced primary/secondary BA ratio (PBA/SBA) in BA pools in the blood of *db*/*db* mice after AGI treatment vs untreated controls (Fig. 1k–m, ESM Fig. 2a and ESM Table 4) was consistent with what has been observed in human participants and what has been linked with improved metabolic parameters [8, 30]. In addition, compared with the control, the known FXR antagonist [31], taurine-conjugated β murine cholic acid (TβMCA), increased in cecum content but decreased in the blood and liver with AGI treatment (Fig. 1n–p), in line with changes in taurine-conjugated cholic acid (TCA)/TβMCA ratio (indicating FXR activation; Fig. 1q–s), suggesting that the FXR was activated in the liver and inhibited in the distal ileum. Accordingly, FXR-targeting genes, such as those encoding cytochrome P450, family 8, subfamily b, polypeptide 1 (CYP8B1) in the liver, which promotes cholic acid synthesis, and fibroblast growth factor 15 (FGF15) in the distal ileum, which inhibits BA synthesis, were both downregulated by AGI vs untreated controls (ESM Fig. 2b). Furthermore, hepatic expression of *Cyp7a1*, which encodes the key enzyme regulating BA synthesis (cytochrome P450, family 7, subfamily a, polypeptide 1 [CYP7A1]) was not different between untreated controls and AGI-treated mice (ESM Fig. 2d), probably due to the converged effect of downregulated gut FGF15 and activated hepatic FXR [15, 31]. In contrast, genes regulating ileum BA reabsorption, such as those encoding the sodium-dependent cholesterol transporter (*Asbt*; also known as *Slc10a2*) and organic solvent tolerance protein β (*Ostβ*; also known as *Slc51b*), were upregulated by AGI (ESM Fig. 2c), indicating increased gut BA reabsorption. Taken together, AGI induced similar changes in gut microbiome and BA compositions in *db*/*db* mice as those observed in humans. We further found that enterohepatic BA circulation, rather than liver BA synthesis, could possibly be more greatly affected by AGI treatment.

### The blood glucose- and triacylglycerol-lowering effect of AGI requires FXR signalling

**Fig. 2:**
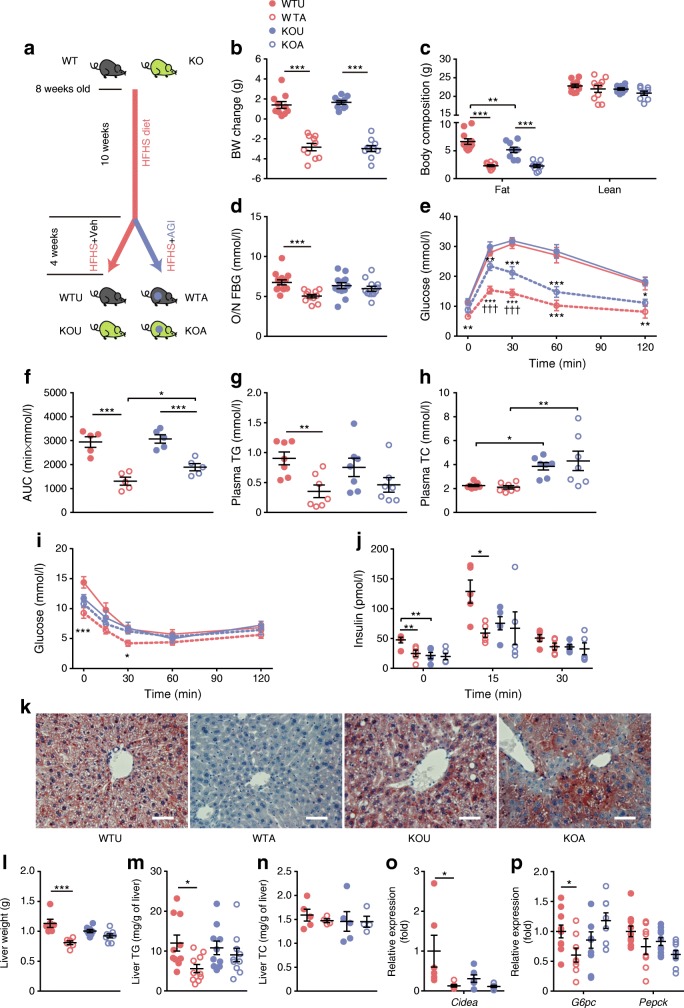
The glucose-lowering effects of AGI were dependent on FXR signalling. (**a**) Schematic of the study design. All mice were fed an HFHS diet. WTU, untreated wild-type mice; WTA, wild-type mice treated with AGI; KOU, untreated *Fxr*-KO mice; KOA, *Fxr*-KO mice treated with AGI. Both KO mice and their WT littermates were fed on an HFHS diet from 8 weeks of age for 10 weeks. Following this, they were treated with AGI or vehicle (Veh) for 4 weeks. (**b**) Body weight (BW) changes after 4 weeks of AGI treatment; *n* = 10 per group. (**c**) Body composition; *n* = 10 per group. (**d**) Overnight (O/N) fasting blood glucose (FBG); *n* = 10 per group. (**e**) IPGTT; *n* = 5 per group. (**f**) AUC of total blood glucose excursions after a glucose bolus; *n* = 5 per group. (**g**, **h**) Plasma triacylglycerol (**g**) and total cholesterol (**h**); *n* = 7–9 per group. (**i**) ITT; WTU, *n* = 9; WTA, *n* = 7; KOU, *n* = 7; KOA, *n* = 7. (**j**) In vivo GIIS test; *n* = 5 per group. (**k**) Representative Oil Red O staining of liver sections in HFHS diet-fed mice after AGI treatment; *n* = 3 per group. Scale bars, 100 μm. (**l**) Liver weight, (**m**) liver triacylglycerol and (**n**) liver total cholesterol in HFHS diet-fed mice after AGI treatment; *n* = 4–10 per group. (**o**, **p**) mRNA expression of genes regulating lipid accumulation (**o**) and gluconeogenesis (**p**); *n* = 4–9 per group. Key in (**b**) also applies to (**c**–**j**) and (**l**–**p**). Data are presented as mean±SEM. *p < 0.05, **p < 0.01, ***p < 0.001 as indicated, except for (**e**) and (**i**), which shows WTU vs WTA /KOU vs KOA; ^†††^*p* < 0.001, WTA vs KOA; all analysed by two-way ANOVA. TG, triacyclglycerols; TC, total cholesterol

To further test if BA signalling was responsible for the metabolic benefits of AGI, we examined the effects of AGI in *Fxr* global KO (*Fxr*- KO) mice on a C57BL/6J background or their wild-type littermates that were fed on an HFHS diet (details of the study design are illustrated in Fig. 2a). Obesity and hyperglycaemia were induced in mice before AGI treatment (ESM Fig. 3a, b). The metabolic improvements induced by AGI treatment of wild-type vs untreated wild-type mice, such as improved fasting blood glucose, glucose tolerance and blood triacylglycerol levels, were attenuated in AGI-treated *Fxr*-KO mice vs untreated KO mice (Fig. 2d–h). Of note, though the effect was lessened, glucose tolerance was still significantly improved in *Fxr*-KO mice treated with AGI compared with untreated mice (Fig. 2e, f). Plasma total cholesterol was different depending on genetic background of the mice (*p* < 0.05 for AGI treated or untreated *Fxr*-KO vs respectively treated/untreated wild-type mice), rather than AGI treatment (*p* > 0.05 for AGI treated wild-type vs untreated wild-type and AGI treated KO vs untreated KO mice) (Fig. 2h), which is in line with previous studies [32, 33]. Further, improved insulin sensitivity (Fig. 2i) along with lower in vivo GIIS (Fig. 2j) with AGI treatment (the ‘so-called’ insulin-sparing effect [8, 29] of AGI) was also notably attenuated in AGI-treated KO mice.

The role of FXR in regulating insulin sensitivity and liver metabolism is well recognised [16, 33]. AGI reduced liver weight and hepatic triacylglycerol accumulation (Fig. 2k–m) with reduced expression of the gene encoding cell death-inducing DNA fragmentation factor alpha-like effector protein a (*Cidea*; Fig. 2o) and reduced gluconeogenesis via inhibition of the genes encoding glucose-6-phosphatase, catalytic subunit (*G6pc*; *p* < 0.05) and phosphoenolpyruvate carboxykinase 1, cytosolic (*Pepck*; also known as *Pck1*; *p* = 0.053) (Fig. 2p); these changes were abrogated in *Fxr*-KO. Thus, metabolic phenotyping suggests that the glucose- and lipid-lowering effects of AGI are partially dependent on FXR activity, which could be due to FXR-dependent improvements in insulin hypersecretion and hepatic metabolism.

### AGI alters BA pool partitioning and signalling in enterohepatic tissues of HFHS diet-fed mice

**Fig. 3:**
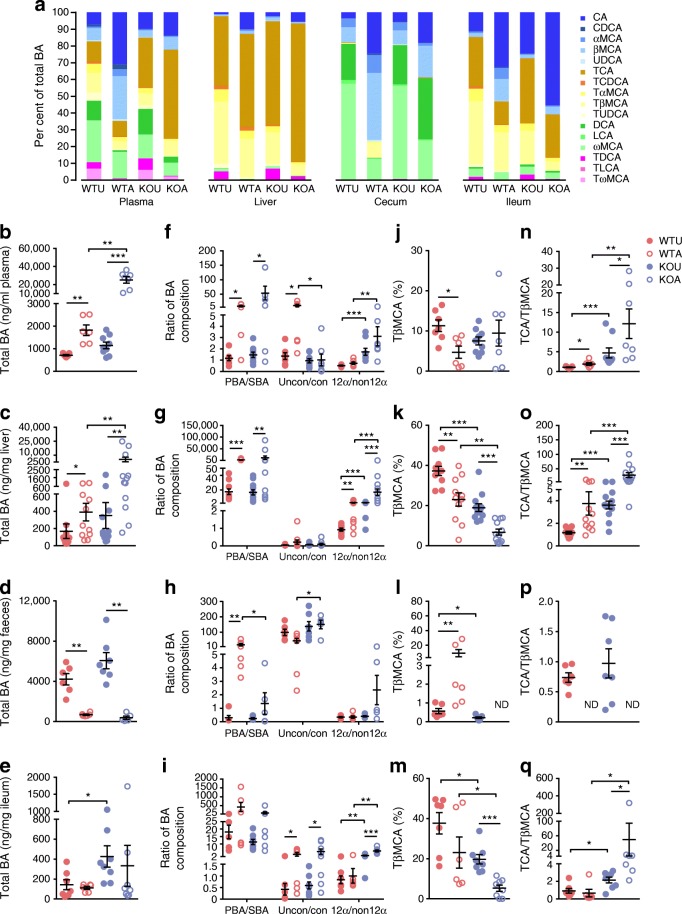
Changes in BA pools in the enterohepatic tissues and circulation after AGI treatment. (**a**) Stacked bar plot of BA pools in plasma, liver, cecum content and ileum of untreated wild-type mice (WTU), wild-type mice treated with AGI (WTA), untreated *Fxr*-KO mice (KOU) and *Fxr*-KO mice treated with AGI (KOA) (statistical results in ESM Table 5). Plasma: WTU, *n* = 7; WTA, *n* = 6; KOU, *n* = 9; KOA, *n* = 7. Liver: WTU, *n* = 9; WTA, *n* = 11; KOU, *n* = 12; KOA, *n* = 12. Cecum content: WTU, *n* = 6; WTA, *n* = 6; KOU, *n* = 7; KOA, *n* = 5. Ileum: WTU, *n* = 7; WTA, *n* = 6; KOU, *n* = 8; KOA, *n* = 8. (**b**–**q**) Changes in BA content and composition in HFHS-fed mice in plasma (**b**, **f**, **j** and **n**), liver (**c**, **g**, **k** and **o**), cecum content (**d**, **h**, **l** and **p**) and ileum (**e**, **i**, **m** and **q**); *n* = 6–12 per group. Key in (**n**) applies to (**b**–**q**). Data are presented as mean±SEM. *p < 0.05, **p < 0.01, ***p < 0.001, Mann–Whitney *U* test. ND, not detected; CA, cholic acid; CDCA, chenodeoxycholic acid; DCA, deoxycholic acid; LCA, lithocholic acid; MCA, muricholic acid; TCDCA, taurochenodeoxycholic acid; TDCA, taurodeoxycholic acid; TLCA, taurolithocholic acid; TαMCA, tauro-α-muricholic acid; TβMCA, tauro-β-muricholic acid; TωMCA, tauro-ω-muricholic acid; TUDCA, tauroursodeoxycholic acid; UDCA, ursodeoxycholic acid

We then sought to characterise the signature of BA pools and alterations in signalling in the HFHS-fed mice treated by AGI. The BA profiles in plasma, liver, ileum and cecum content of mice were assayed (Fig. 3a, ESM Table 5) and significantly increased plasma and liver BA pool sizes and downregulated cecum pool sizes were observed with AGI treatment vs untreated controls (Fig. 3b–e). These changes were similar but to a greater extent than those observed in *db*/*db* mice (Fig. 1h–j). The PBA/SBA ratio in blood, liver and cecum content were all increased by AGI treatment, whilst the 12α-hydroxylated/non-12α-hydroxylated BA ratio was only increased in the liver but not in other compartments vs controls. In addition, the unconjugated/conjugated BA ratio in the blood and ileum were increased upon AGI treatment vs controls (Fig. 3f–i); this trend was also observed in the liver of HFHS-fed mice but did not reach significance (Fig. 3g). Also, like in *db*/*db* mice, the changes in the proportion of TβMCA (Fig. 3j–m) and the ratio of TCA/TβMCA (Fig. 3n–q) in BA pools of different compartments were consistent with those in *db*/*db* mice (Fig. 1n–s), i.e. the TCA/TβMCA was enhanced in the blood and liver but was lower (non-detectable) in cecum content with AGI treatment vs controls (Fig. 3n–q). Compared with controls, *Fxr*-KO mice showed similar changes in BA pool as wild-type mice following treatment with AGI, further supporting the notion that the alterations in microbial BA metabolism following treatment with AGI might underlie the AGI-induced BA pool repartition.

**Fig. 4:**
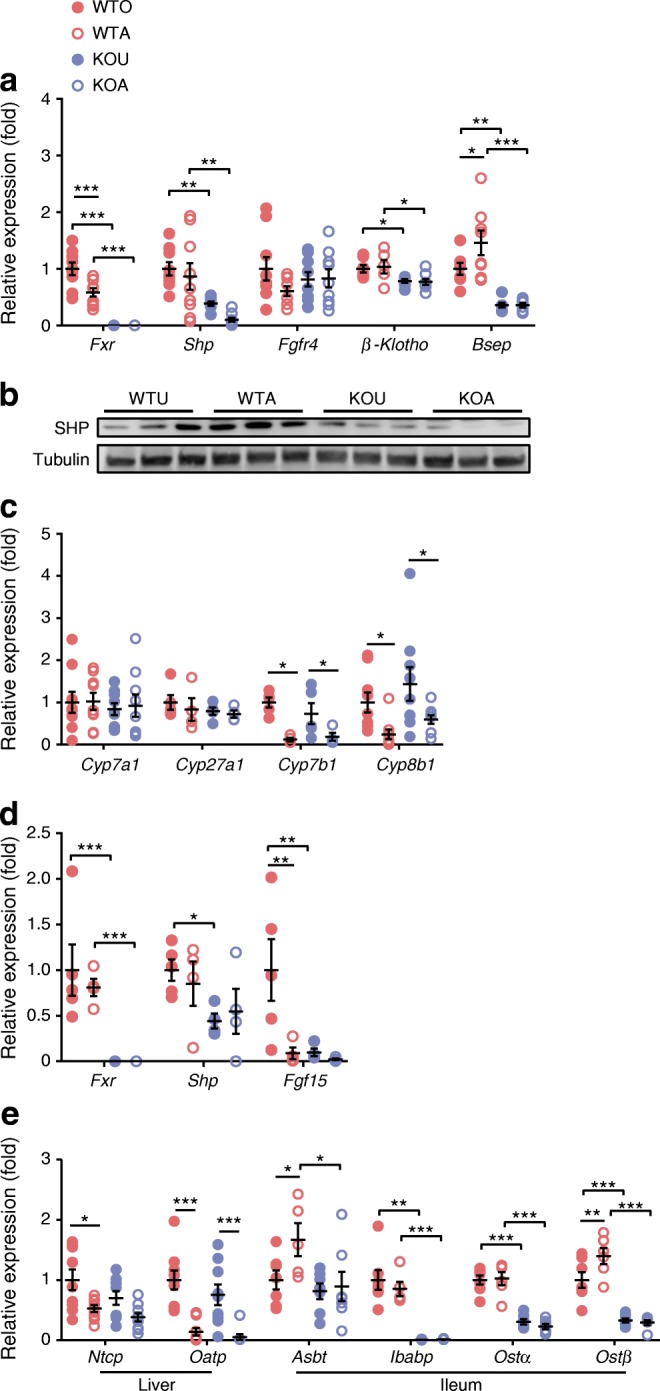
AGI altered enterohepatic FXR signalling and BA metabolism in HFHS-fed mice. (**a**) Relative mRNA expression of FXR-signalling genes in the liver after AGI treatment; *n* = 7–9 per group. Note: *β-Klotho* is also known as *Klb*. KOA, *Fxr*-KO mice treated with AGI; KOU, untreated *Fxr-*KO mice; WTA, wild-type mice treated with AGI; WTU, untreated wild-type mice. (**b**) Western blot analysis of liver SHP; *n* = 3. (**c**) Changes in relative expressions of genes regulating liver BA synthesis. (**d**) Relative mRNA expression of FXR-signalling in the ileum after AGI treatment; *n* = 4–5 per group. (**e**) Expression of BA transportation genes in the liver and ileum; *n* = 5–7 per group. Note: *Ibabp* is also known as *Fabp6*; *Ostα* is also known as *Slc51a*. Key in (**a**) also applies to (**c**–**e**). Data are presented as mean±SEM. *p < 0.05, **p < 0.01, ***p < 0.001, two-way ANOVA

We then examined FXR/BA signalling in the enterohepatic tissues. Similar to the findings in *db*/*db* mice, AGI treatment exerted diverse effects on hepatic and ileum FXR activity and impacted on host BA transportation. We found elevated transcription of the gene encoding bile salt export protein (*Bsep*; also known as *Abcb11*) and protein levels of small heterodimer partner (SHP), and decreased transcription of *Cyp8b1* and the gene encoding cytochrome P450, family 7, subfamily b, polypeptide 1 (*Cyp7b1*) in liver (Fig. 4a–c), suggesting activated hepatic FXR. Of note, the expression of *Cyp7a1*, the gene encoding key BA synthesis enzyme, remained unaltered in the liver following AGI treatment (Fig. 4c). Meanwhile, expression of downstream signals of FXR, *Shp* (also known as *Nr0b2*) and *Fgf15*, were diminished in the distal ileum (Fig. 4d). The BA reabsorption genes *Asbt* and *Ostβ* were increased in the ileum, whilst genes encoding the BA importers sodium taurocholate cotransporting polypeptide (*Ntcp*; also known as *Slc10a1*) and organic anion transporting polypeptide (*Oatp*; *Slco1a1*) were decreased (Fig. 4e) and the expression of a BA exporter (*Bsep*) was enhanced (Fig. 4a) in the liver after AGI treatment. Deletion of *Fxr* nearly ablated all the molecular changes observed with AGI treatment, except for those of *Cyp8b1*, *Cyp7b1* and *Oatp*.

From these findings, we proposed that the BA pool repartitioning after AGI treatment, i.e. decreased biotransformation of PBA to SBA, altered TCA/TβMCA in enterohepatic tissues, enhanced gut BA reabsorption and enlarged BA pool size in the liver and circulation, may alter BA signalling in the liver and ileum (via inhibition of FXR in the gut and activation of FXR in the liver), exerting opposite effects on hepatic BA synthesis, but similar effects with regard to improving metabolic homeostasis [16, 34, 35].

### FXR mediates the effect of AGI on preservation of beta cell function by decelerating beta cell proliferation

**Fig. 5:**
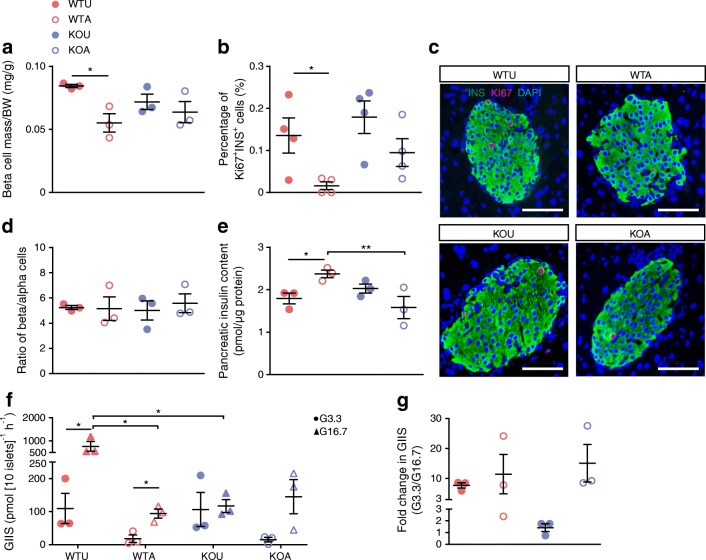
AGI treatment improved beta cell preserve and alleviated insulin hypersecretion via FXR. (**a**) Beta cell mass in HFHS-fed untreated wild-type mice (WTU), HFHS-fed wild-type mice treated with AGI (WTA), HFHS-fed untreated *Fxr*-KO mice (KOU) and HFHS-fed *Fxr*-KO mice treated with AGI (KOA); *n* = 3 per group. (**b**) Percentage of Ki67/insulin double-positive beta cells in insulin-positive beta cells; *n* = 4 per group. (**c**) Representative images of the Ki67 (red) and insulin (green) immunolabelling in pancreas sections. Nuclei stained with DAPI are shown in blue. Scale bars, 100 μm. (**d**) Ratio of insulin-/glucagon-positive (beta/alpha) cells; *n* = 3 per group. (**e**) Quantification of pancreatic insulin content; *n* = 3 per group. (**f**, **g**) Absolute value (**f**) and fold change (**g**) of ex vivo GIIS. Islets were cultured with low glucose (3.3 mmol/l; G3.3) or high glucose (16.7 mmol/l; G16.7). *n* = 3 per group. Key in (**a**) also applies to (**b**, **d**, **e** and **g**). In (**f**): red closed circle, WTU+G3.3; red closed triangle, WTU+G16.7; red open circle, WTA+G3.3; red open triangle, WTA+G16.7; blue closed circle, KOU+G3.3; blue closed triangle, KOU+G16.7; blue open circle, KOA+G3.3; blue open triangle, KOA+G16.7. Data are presented as mean±SEM, *p < 0.05, **p < 0.01, two-way ANOVA. BW, body weight; INS, insulin

We went on to investigate whether FXR also mediated the insulin-sparing effect of AGI. In AGI-treated wild-type mice, we found significantly reduced beta cell mass vs untreated wild-type controls (Fig. 5a) and decreased beta cell proliferation, manifesting as reduced percentage of Ki67-labelled beta cells (Fig. 5b–c). No changes in pancreatic islet structure (ESM Fig. 4a) and the ratio of beta/alpha cells were found with AGI treatment (Fig. 5d) vs untreated controls. Pancreatic insulin content showed a minor but significant elevation after AGI treatment (Fig. 5e). On the other hand, though in vivo treatment of AGI decreased levels of ex vivo insulin secretion under high glucose conditions in primary islets (basal insulin secretion showed a trend of decrement with no significance) (Fig. 5f), it did not affect ex vivo GIIS responses (Fig. 5g) as compared with islets from untreated wild-type mice. However, the AGI-related islet changes were nearly all absent in AGI-treated *Fxr*-KO mice, suggesting a role for islet FXR signalling in regulating the adaptation of the endocrine pancreas in response to nutrient overload. Notably, AGI rescued islet structure and insulin levels in *db*/*db* mice (ESM Fig. 4b,c), with similar effects of decreasing beta cell proliferation (ESM Fig. 4d,e) along with recovery of beta cell MafA and UCN3 levels (key proteins that preserve the mature function of beta cells; ESM Fig. 4f–i).

Though not being able to acquire data on how beta cells proliferate in human participants, we measured beta cell function in ten AGI-treated individuals with type 2 diabetes during a two-step hyperglycaemic clamp (all participants had enrolled onto a clinical trial [ClinicalTrials.gov registration no. NCT01758471]; details are provided in the ESM Methods and ESM Table 6). Despite mitigated insulin secretion response during the oral glucose tolerance test (ESM Table 6), the clamp data showed a better acute insulin secretion response to a meal test post vs pre-treatment (ESM Table 7, ESM Fig. 5) [8], although this was not significant (*p* = 0.077). Overall, the downregulated beta cell mass, proliferation and absolute insulin secretion with AGI treatment may be beneficial to beta cell preserve in diabetes and may also require FXR signalling.

### Transcriptome profiling discovered genes regulating beta cell proliferation targeted by AGI are dependent on FXR

**Fig. 6:**
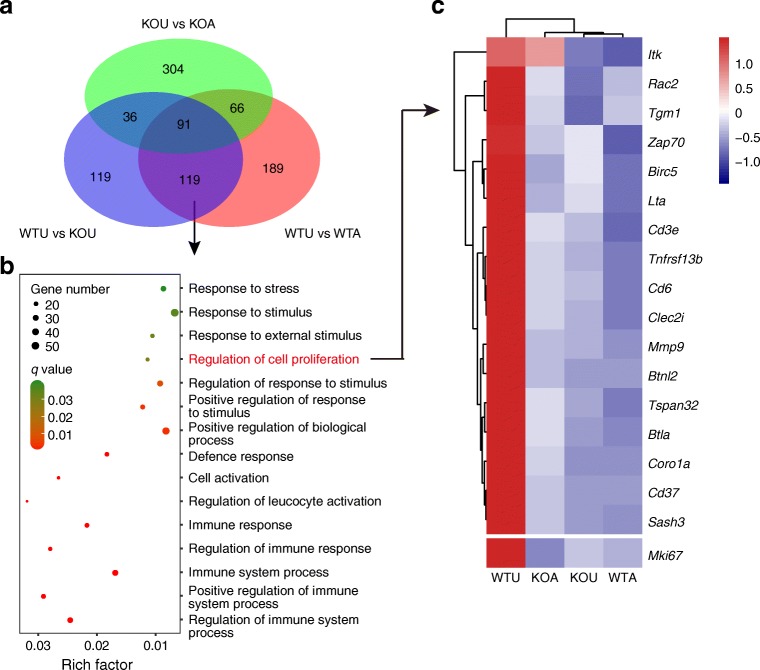
Analysis of RNA sequencing data of primary islets. (**a**) Venn diagram of overlapping DEGs among untreated wild-type mice (WTU), wild-type mice treated with AGI (WTA), untreated *Fxr*-KO mice (KOU) and *Fxr*-KO mice treated with AGI (KOA). One hundred and nineteen FXR-dependent AGI-regulated genes were discovered. (**b**) Top 15 most enriched GO terms in the biological process (BP) term (*q* < 0.05) of the 119 FXR-dependent AGI-regulated genes. The *x*-axis represents the enrichment factor (calculated by the number of DEGs/number of GO term genes) and the *y*-axis presents GO terms. The colour of the dots indicates the *q* value (high [green] to low [red]). The dot size indicates the number of DEGs contained in each GO term. (**c**) Heatmap of 17 genes in the GO for regulation of cell proliferation and the gene *Mki67*. Colour key shows the *z*-score of normalised relative expression of genes in each row. *n* = 3 per group

To further understand the mechanism underlying the FXR-dependent response of pancreatic islets to AGI treatment, we performed RNA sequencing on isolated primary islets from different mouse groups. In total, there were 465 differentially expressed genes (DEGs) that were induced by AGI treatment in wild-type mice vs untreated wild-type mice and 497 in *Fxr*-KO mice vs untreated *Fxr*-KO mice (fold change ≥2.0, *q* ≤ 0.001) and only 157 genes overlapped in these two comparisons, as shown in the Venn diagram in Fig. 6a. We hypothesised that the genes overlapping between DEG gene sets in untreated vs AGI-treated wild-type mice and untreated wild-type vs untreated *Fxr*-KO mice, excluding those that were different between untreated vs AGI-treated *Fxr*-KO mice, were those targeted by AGI and dependent on FXR activity. In total 119 DEGs of this kind were further analysed using gene ontology (GO) enrichment (Fig. 6b). Regulation of cell proliferation was among the 15 most significantly annotated GO terms (Fig. 6c). The 17 DEGs in this GO were all downregulated after AGI treatment in wild-type mice and included the gene for baculoviral inhibition of apoptosis protein (IAP) repeat-containing 5 (*Birc5*), a gene encoding survivin, which is known for its role in promoting pregnancy- and surgery-induced beta cell proliferation [36, 37]. Though not included in the annotation of cell proliferation, expression of *MKi67* (gene encoding Ki67) was also significantly decreased after AGI treatment in wild-type mice and did not change in *Fxr*-KO mice (Fig. 6c), which paralleled with the Ki67 immunostaining results in islets (Fig. 5b). However, though genes regulating insulin secretion biosynthesis (i.e. *Ins1* and *Ins2*), exocytosis (i.e. *Slc30a8* and *Slc2a2*) and beta cell identity (i.e. *Mafa*, *Foxa2* and *Pdx1*) were not assigned as DEGs (fold change <2.0), they were slightly but significantly altered by AGI treatment in wild-type mice vs untreated mice (*q* ≤ 0.001; ESM Fig. 6a) [38–40]. How these changes may affect beta cells in the long term awaits further study. Islet immunolabelling of MafA showed no obvious changes between the mouse groups (ESM Fig. 6b). In addition, FXR protein levels were comparable in wild-type mice treated with and without AGI and absent in treated and untreated *Fxr*-KO mice (ESM Fig. 6c). Thus, the transcriptome analysis suggested that the AGI-induced inhibition of the cell proliferation signalling pathway in pancreatic islets might lead to decelerated beta cell hyperplasia and cell mass expansion in an FXR-dependent manner.

## Discussion

Tremendous interest has been aroused in studying the crosstalk of gut commensal microbiota and its role in regulating host health. In this study, by mainly employing *Fxr*-KO mice fed an HFHS diet, we confirmed that the glucose- and lipid- lowering effects of AGI were partially mediated by its effect on gut microbial BA metabolism and, hence, host BA signalling. In addition to slowing down glucose absorption, altered BA signalling in the gut, liver and beta cell upon treatment may synergistically contribute to the glucose-lowering effects of AGI via improving insulin resistance and beta cell function preserve. We further characterised BA pool alterations after AGI treatment and studied related signalling in both enterohepatic tissues and pancreatic islets. Our study delineated a potential BA-dependent regulation of AGI treatment effects in multiple key metabolic organs, which contributed to its glucose-lowering effects and metabolic benefits.

First, after comparing the BA profile in multiple organ compartments, the AGI-induced regulation of BA pools might be characterised by reduced microbial BA production via inhibition of PBA biotransformation, which was more easily reabsorbed from the gut due to enhanced ileum *Asbt* and *Ostβ* expression. This change eventually led to expanded BA pool sizes, increased PBA portion and decreased gut BA excretion in the host liver and circulation. The opposing activation status of FXR in the liver and ileum might have led to the unaltered *Cyp7a1* expression after AGI treatment, which suggests that the BA pool size expansion might not be due to increased BA synthesis. Of note, CYP8B1, the key enzyme that controls cholic acid synthesis, was significantly decreased by AGI. Both activated hepatic FXR and increased insulin signalling after AGI treatment can inhibit CYP8B1 [30, 41–43]. Moreover, FGF15 exerts less inhibitory effect on CYP8B1 than CYP7A1 [42, 43]. *Cyp8b1*-KO mice or mice with inhibited liver CYP8B1 [30, 44] have shown improved insulin sensitivity and glucose metabolism. Thus, we thought the consistent decreased expression of *Cyp8b1* after treatment with AGI may not influence AGI-associated alterations in BA but may be linked to changes in insulin signalling and metabolic benefits. However, the 12α-/non-12α-hydroxylated BA ratio was not decreased with the reduction in *Cyp8b1*, and was even increased in the liver BA pool. We assume that the altered intestinal BA reabsorption and microbial PBA biotransformation induced by AGI might override the impact of CYP8B1 on the regulation of 12α-/non-12α- hydroxylated BA ratio, and this hypothesis surely awaits further investigation.

The AGI-induced BA changes brought about diverse BA signal regulation in different organs, inducing organ-specific regulation. Use of organ-specific *Fxr*-KO mice could help to elucidate the compartment in which altered FXR signalling mainly mediates the glucose-lowering effects of AGI. However, activated hepatic and inhibited intestinal BA signalling, for example, have both been proven to improve metabolism status [16, 34, 35, 45]. Thus, strategies other than simply using organ-specific *Fxr*-KO animals might be required to pinpoint the main tissue that mediates BA-dependent AGI treatment effects.

There is increasing evidence that insulin hypersecretion can be the cause, rather than the consequence, of peripheral insulin resistance, which initiates hyperglycaemia [46–49]. Recently, using single-cell sequencing, a study has confirmed that increased beta cell replication is linked to functional immaturity of these cells [50]. Thus, the ability for AGI to reduce cell proliferation without affecting the GIIS response could prevent further loss of beta cell preserve, acting as a ‘brake’ for beta cell compensative proliferation, and might lead to long-term glycaemic control [51–53]. The recovered islet structure, function and key pancreatic transcription factor expression in *db*/*db* mice and the insulin-sparing effect in humans with AGI treatment further supports the notion that the negative effect of AGI on beta cell proliferation might be beneficial in the preservation of beta cell function in diabetes.

BA signalling has been related to benign and malignant cell proliferation in enterohepatic tissues under different scenarios [54, 55]. Hence, the new observation of *Fxr*-mediated AGI regulation of beta cell proliferation is conceivable. Our islet RNA sequencing results also demonstrate that genes involved in cell proliferation, such as *Birc5* and *Ki67*, were significantly decreased with AGI treatment, which was dependent on FXR activity. There were no significant changes in islet lipotoxicity-related genes after AGI treatment (ESM Table 8), which may lessen the possibility that the impact of AGI treatment on islets could be derived from its FXR-dependent hypolipidaemic effects.

Of note, a recent study showed that transplanting faeces from AGI-treated individuals with diabetes did not lower blood glucose levels in high-fat-diet (HFD)-fed germ-free mice [56]. Consistently, we found that pre-treatment of mice with an antibiotic mix as a proxy for germ-free status did not attenuate the glucose-lowering and bodyweight-reducing effects of AGI (ESM Fig. 7). Hence, the easy conclusion can be drawn that the glucose-lowering effects of AGI are not mediated by gut microbiota. However, it should be noted that ablating the gut microbiome either in germ-free mice or mice treated with an antibiotic mix leads to an extraordinary enlarged BA pool and altered BA components, resulting in altered FXR signalling, and contributes to a phenotype that resists HFD-induced metabolic disorders [18]. The extremely altered FXR signalling in germ-free mice could override the changes brought about by AGI treatment, also changing microbial BA metabolism. Hence, the current methodology of using germ-free mice to confirm the causal correlation between alterations in microbiota and host metabolic health might not always be appropriate, particularly in the scenario where BA signalling is required for relaying microbiota cues to the host.

In summary, we characterised BA pool and BA signalling alterations in mouse models of diabetes upon treatment with AGI. More importantly, with the aid of *Fxr*-KO mice, we confirmed that AGI at least partially exerts its therapeutic effects on metabolism by orchestrating BA signalling in multiple organs, including the endocrine pancreas (ESM Fig. 8). Our study, thus, sheds light on the significance of bacterial BA metabolism in regulating host metabolism and its potential as a new target for the treatment of type 2 diabetes.

## Electronic supplementary material

ESM (PDF 1.32 mb)

## Data Availability

All the supporting data are in the ESM. The sequencing data have been uploaded onto the BioProject Database (accession no. PRJNA600345; www.ncbi.nlm.nih.gov/bioproject/600345).

## Abbreviations

AGI: α-Glucosidase inhibitor
BA: Bile acid
CYP7A1: Cytochrome P450, family 7, subfamily a, polypeptide 1
CYP8B1: Cytochrome P450, family 8, subfamily b, polypeptide 1
DEG: Differentially expressed gene
FGF15: Fibroblast growth factor 15
FXR: Farnesoid X receptor
GIIS: Glucose-induced insulin secretion
GO: Gene ontology
HFD: High-fat diet
HFHS: High-fat high-sucrose
KO: Knockout
PBA: Primary bile acid
SBA: Secondary bile acid
TCA: Taurine-conjugated cholic acid
TβMCA: Taurine-conjugated β murine cholic acid
UCN3: Urocortin-3
UPLC: Ultra-performance liquid chromatography
