# Supplementary material for: The glucose-lowering effects of α-glucosidase inhibitor require a bile acid signal in mice
Source: Diabetologia. 2020 Feb 8;63(5):1002–16. doi: 10.1007/s00125-020-05095-7 (PMC7145781; doi:10.1007/s00125-020-05095-7)
Supplement: Supplementary file 1 — (PDF 1.32 mb) [file 125_2020_5095_MOESM1_ESM.pdf]

ESM methods

Blood glucose and insulin tolerance tests

After 6 hours fasting (from 8:00 hours to 14:00 hours), 22-week-old *Fxr* knockout mice and their littermates were subjected to intraperitoneal glucose tolerance tests (IPGTT), *in vivo* glucose-induced insulin secretion (GIIS) and insulin tolerance tests (ITT). Mice were injected intraperitoneally with 2 mg dextrose/kg body weight or 0.75 IU insulin/kg body weight (Novo Nordisk, Princeton, NJ). Blood glucose concentrations were measured by glucometers (Roche, Basel, Switzerland). Serum insulin concentrations were assayed by UltraSensitive Mouse Insulin ELISA kit (Alpco, Salem, New Hampshire, USA) via great saphenous bleeding.

16S rRNA gene sequencing of the gut microbiota and quantification of Bacterial DNA

Bacterial DNA extraction of mouse colon content was followed as previously reported [1] and then subjected to PCR amplification and Illumina MiSeq sequencing. The v3-v4 region of the bacteria 16S ribosomal RNA gene was amplified using primers 341F, ACTCCTACGGGAGGCAGCAG and 806R, GGACTACHVGGGTWTCTAAT. The qualified libraries were sequenced pair end on the Miseq system (Illumina, San Diego, California, USA). The clean reads were obtained as previous studies [2]. And tags were clustered to Operational Taxonomic Units (OTU) at 97% sequence similarity. OTU representative sequences were taxonomically classified using Ribosomal Database Project (RDP) Classifier v.2.2 trained on the Greengenes database, using 0.6 confidence values as cut off. At last, alpha diversity, beta diversity and the different species screening were analysed based on OTU and taxonomic ranks.

Real-time PCR amplification and detection was performed using the SYBR Green II Master (Takara, Kusatsu, Japan) on LightCycler 480 (Roche Applied Science, Indianapolis, USA), with the primers of total bacteria (UniF340, ACTCCTACGGGAGGCAGCAGT and UniR514, ATTACCGCGGCTGCTGGC) [3]. Quantification of bacterial DNA was followed as previously reported [4].

Triglycerides and cholesterol measurement

Plasma triglyceride (TG) and total cholesterol (TC), hepatic TG and TC in mice were measured by Quantification Colorimetric/Fluorometric Kit (Biovision, Milpitas, California, USA) according to the manufacturer's instructions. For hepatic lipids assay, liver tissues were homogenized in Folch solution (chloroform/methanol, 2:1, v/v) and the tissue lysates were extracted for further analysis.

Pancreatic beta cell mass measurement

Pancreas were carefully removed from mice, weighed and then embedded and sectioned continuously. 9-10 evenly 200 μm apart sections throughout the entire pancreas were analysed as previously described [5]. Digital images of sections were captured by ZEISS Axio Scan.Z1 Slide Scanner (Zeiss, Oberkochen, Germany). Total pancreatic and insulin-positive areas of each section were measured using Meta-Morph v7.1 software (Molecular Devices, Sunnyvale, CA USA).

Pancreatic insulin content

Whole pancreatic tissue of mice was removed and weighed. Each pancreas was homogenized in acidic ethanol and rotated at 4℃ for 48 hours and then centrifuged to collect the supernatant. The insulin level of the supernatant was determined by ELISA assay (Mouse Insulin ELISA kit, Alpco, Salem, New Hampshire, USA) and the total protein level was assayed

by BCA Protein Assay Kit (Thermo Fisher Scientific, Waltham, MA, USA).

Islet isolation and *ex vivo* GIIS assay

Mice pancreas were injected with collagenase P (Roche, Basel, Switzerland) via the common hepatic bile duct and then digested at 37℃ water bath, islets were then hand-picked under stereoscope [5]. Picked islets were either collected to snap-frozen in liquid nitrogen for RNA/Protein extraction or subjected to *ex vivo* batch GSIS. Islets were cultured in RPMI 1640 with 10% FBS (Thermo Fisher Scientific) for 4 hours and then islets were switched to Krebs-Ringer HEPES buffer (KRB; 120 mmol/l NaCl, 4.8 mmol/l KCl, 2.5 mmol/l $CaCl_2$, 1.2 mmol/l $MgSO_4$, 1.2 mmol/l KH_2P0_4, 10 mmol/l $NaHCO_3$) containing low glucose (3.3 mmol/l) or high glucose (16.7 mmol/l) for 1 hours. After supernatant was collected for insulin assay. The islets were lysed with acidic ethanol and subjected to further insulin assay using Mouse Insulin ELISA kit (Alpco).

Immunolabelling assay

Immunolabelling and image processing procedures were performed as previously described [5]. Antibodies were validated by utilizing negative control slides (primary or secondary antibody excluded). Fluorescence images were captured with OLYMPUS BX51 microscope (OLYMPUS, Tokyo, Japan). Quantification of Glucagon-positive, MafA-positive and Ki67-positive cell number, and Ucn3 fluorescence intensity were performed with ImageJ v1.48 (National Institutes of Health, Bethesda, MD, USA). At least 2000 insulin-positive cells or 50 islets from each mouse were counted for quantification. Primary antibodies and immunofluorescence secondary antibodies were diluted in DakoCytomation antibody diluent (Dako, Burlington, ON, Canada) at the reported dilution in ESM table 2.

Real-time quantitative RT-PCR

Total tissue RNA was extracted by TRIzol reagent (Invitrogen, Waltham, MA, USA) in accordance with the manufacturer's protocols. Total islet RNA was extracted using RNeasy Plus Micro kit (Qiagen, GmBH, Germany) and reverse transcription with the Reverse Transcription System Kit (Promega, Madison, USA) to cDNA. Real-time PCR amplification and detection were performed using the SYBR Green II Master (Takara, Kusatsu, Japan) on LightCycler 480 (Roche Applied Science, Indianapolis, USA). The sequences of all primers used in this study were listed in ESM Table 3. The expression levels were normalized to housekeeping *36b4* (also known as *Rplp0*) gene.

Western blotting and antibodies

Proteins were extracted from tissues in mice with RIPA buffer (Cell Signaling Technology, Beverly, MA, USA) and quantified using BCA Protein Assay Kit (Thermo Fisher Scientific). Proteins were separated by SDS-PAGE, subjected to electrophoretic transfer on nitrocellulose membranes and then blotted with primary antibodies, the blotting bands were illuminated by Immobilon Western Chemiluminescent HRP (Merck Millipore, Darmstadt, Germany) and captured with Image Quant LAS 4000 (GE Healthcare Life Sciences, Boston, USA). Primary antibodies and secondary antibodies were diluted in Tris-Buffered Saline Tween-20 (TBST) buffer containing 5% (wt/vol) Bovine Serum Albumin (BSA). Antibodies were validated by utilizing negative control (primary or secondary antibody excluded). Antibodies information was listed in ESM Table 2.

RNA Sequencing of pancreatic islets

The extracted RNA from isolated mice primary islet samples were subjected to library

construction and sequencing based on BGISEQ-500 platform [6]. RNA-seq raw data were initially filtered to obtain clean data after quality control, including removing adaptors, reads with more than 5% unknown bases and low-quality reads. Clean data were aligned to the mouse genome (mm10) by HISAT2 v2.0.4 software (The Johns Hopkins University, Baltimore, Maryland, USA). For gene expression analysis, DEGseq, a package in R software (v3.1.1), was using to detect differential expression genes (DEGs) [7, 8]. The significance of DEGs was defined by the combination of the absolute fold change ⩾ 2 and q value ⩽ 0.001 based on the poisson distribution. Functional pathway analysis of DEGs was performed by using phyper, a function of R software (v3.1.1). Z-score was calculated to normalize DEGs among each group by R software. Z-score: $z=(x-\mu)/\sigma$, x=relative expression of each gene, μ=mean of x, σ=standard deviation of x.

Two-step Hyperglycaemic clamp on human type 2 diabetes subjects

Two-step hyperglycaemic clamp was employed to evaluate beta cell function in drug naive type 2 diabetes patients who received AGI treatment [9]. The present study included the patients who participated in a 3-month intervention study to examine the antidiabetic effects of AGI as well as the gut microbiota disturbing effects registered in ClinicalTrials.gov. NCT01758471) [10]. The trial conformed to the provisions of the Declaration of Helsinki and was approved by the ethics committees at each participating centre. Ten of the patients treated by AGI agreed to receive and complete the hyperglycaemic clamp before treatment and 9 after treatment with consent form assigned. Two-step hyperglycaemic clamp were performed as previous described [9]. Briefly, the patients were subjected to the test in the early morning after overnight fasting. The blood glucose levels were stringently maintained around 14 mmol/l by monitoring blood glucose and continuous 20% glucose infusion at average rate of 6 mmol/l/KgBW/min via venous followed by a bolus of 50% glucose. The experiment lasted for at least 5 hours and was composed of two consecutive steps, first venous step (Step I, the hyperglycaemic clamp experiment following a venous glucose bolus) and then oral step (Step II, a second course of hyperglycaemic clamp following a carbohydrate meal). The oral step is used to appreciate the potential of incretin stimulated insulin secretion. Blood samples were taken every 2 min during Phase 1 and every 10 min during Phase 2 (ESM Table 6 and ESM Fig. 5). Serum insulin and C-peptide were measured by chemiluminescence (Roche Diagnostics Corp., Indianapolis, In, USA)

Antibiotic pre-treatment

For antibiotics treatment, mice were randomly divided into db and db Abx group upon purchase. The db group were subjected to only drinking water throughout the experiment, and the Abx group treated with antibiotic cocktail containing ampicillin (1 g/l), neomycin (1 g/l), streptomycin (1 g/l) and doxycycline (1 g/l) in the drinking water for 12 days before AGI treatment. Antibiotics were purchased from Sigma Aldrich, St. Louis, USA.

ESM Table 1. Diet formulas

**D10012G (chow diet for *db/db* mice)**	**g%**	**kJ%**
Protein	20	20
Carbohydrate	64	64
Fat	7	16
Total		100
kJ/g	16.736	
Ingredient	g	kJ
Casein	200	3347.2
L-Cystine	3	50.2
Corn Starch	397.486	6652.6
Maltodextrin 10	132	2209.2
Sucrose	100	1673.6
Cellulose, BW200	50	0
Soybean Oil	70	2635.9
t-Butylhydroquinone	0.014	0
Mineral Mix S10022G	35	0
Vitamin Mix V10037	10	167.4
Choline Bitartrate	2.5	0
Dye, Red	0	0
Dye, Yellow	0	0
Total	1000	16736
**D12266B (HFHS diet)**	**g%**	**kJ%**
Protein	16.8	16.8
Carbohydrate	51.4	51.4
Fat	31.8	31.8
Total		100
kJ/g	18.451	
Ingredient	g	kJ
Casein, 30 Mesh	190	3179.8
DL-Methionine	3	50.2
Corn Starch	215	3598.2
Maltodextrin 10	75	1255.2
Sucrose	290	4853.4
Cellulose, BW200	30	0
Butter Fat, Anhydrous	44.2	1664.4
Corn Oil	118	4443.4
Mineral Mix S10001	40	0
Calcium Carbonate	5.5	0
Sodium Chloride	5.5	0
Potassium Citrate, 1 H2O	13.5	0
Vitamin Mix V10001	11	184.1
Choline Bitartrate	2	0
FD&C Yellow Dye #5	0	0

FD&C Red Dye #40	0.1	0
Total	1042.8	19233

ESM Table 2. Antibodies for Immunolabelling assay and Western blot analysis

Antibody	Dilution (vol/vol)	Source	Catalogue	RRID
guinea pig anti-insulin	1:200[A]	Abcam, Cambridge, MA, USA	ab7842	AB_306130
mouse anti-Glucagon	1:400[A]	Abcam, Cambridge, MA, USA	ab10988	AB_297642
rabbit anti-MafA	1:1000[A]	Bethyl, Montgomery, TX, USA	IHC-00352	AB_1279486
rabbit anti-Ucn3	1:400[A]	Phoenix Pharmaceuticals, Belmont, California, USA	H-019-29	N/A
rabbit anti-Ki67	1:400[A]	Bethyl, Montgomery, TX, USA	IHC-00375	AB_1547959
Alexa Fluor® 488 AffiniPure Donkey Anti-Guinea Pig IgG (H+L)	1:500[A]	Jackson Immunoresearch, West Grove, PA, USA	706-545-148	AB_234472
Alexa Fluor® 594 AffiniPure Goat Anti-Rabbit IgG (H+L)	1:500[A]	Jackson Immunoresearch, West Grove, PA, USA	111-585-003	AB_2338059
Alexa Fluor® 594 AffiniPure Donkey Anti-Mouse IgG (H+L)	1:500[A]	Jackson Immunoresearch, West Grove, PA, USA	715-585-150	AB_2340854
rabbit anti-Shp	1:1000[B]	Abcam, Cambridge, MA, USA	ab186874	AB_2797389
mouse anti-Fxr	1:1000[B]	R&D Systems, Minneapolis, MN, USA	PP-A9033A-00	AB_1962505
rabbit anti-TUBULIN	1:5000[B]	Cell Signaling Technology, Boston, MA, USA	#2144	AB_2210548
Anti-rabbit IgG, HRP-linked Antibody	1:2000[B]	Cell Signaling Technology, Boston, MA, USA	#7074	AB_2099233
Anti-mouse IgG, HRP-linked Antibody	1:2000[B]	Cell Signaling Technology, Boston, MA, USA	#7076	AB_330924

[A]Dilution selected for Immunolabelling assay.

[B]Dilution selected for Western blot analysis.

ESM Table 3. RT-PCR primers

Gene symbol	Forward primer	Reverse primer
*36b4**	GAAACTGCTGCCTCACATCCG	GCTGGCACAGTGACCTCACACG
Asbt	GTCTGTCCCCCAAATGCAACT	CACCCCATAGAAAACATCACCA
Bsep	TGGTGATTCTCGCTGTCAGT	GCAGCCACTGTTCGAATAGA
Cidea	TGACATTCATGGGATTGCAGAC	GGCCAGTTGTGATGACTAAGAC
Cyp27a1	CCAGGCACAGGAGAGTACG	GGGCAAGTGCAGCACATAG
Cyp7a1	AGCAACAACAACCTGCCAGTACTA	GTCCGGATATTCAAGGATGCA
Cyp7b1	GGAGCCACGACCCTAGATG	TGCCAAGATAAGGAAGCCAAC
Cyp8b1	CCTCTGGACAAGGGTTTTGTG	GCACCGTGAAGACATCCCC
Fgf15	GCCATCAAGGACGTCAGCA	CTTCCTCCGAGTAGCGAATCAG
Fgfr4	TTTCTAGTTCCCCCAAAACCTAG	ACACCAGAGCTGATGCCCCTTT
Fxr	CCTGAGAACCCACAGCATTT	GTGTCCATCACTGCACATCC
G6pc	CGACTCGCTATCTCCAAGTGA	GGGCGTTGTCCAAACAGAAT
Ibabp	CTTCCAGGAGACGTGATTGAAA	CCTCCGAAGTCTGGTGATAGTTG
Ntcp	ATCTGACCAGCATTGAGGCTCT	CCGTCGTAGATTCCTTTGCTGT
Oatp	CAGTCTTACGAGTGTGCTCCAGAT	ATGAGGAATACTGCCTCTGAAGTG
Ostα	TGTTCCAGGTGCTTGTCATCC	CCACTGTTAGCCAAGATGGAGAA
Ostβ	CAGTCTTACGAGTGTGCTCCAGAT	ATGAGGAATACTGCCTCTGAAGTG
Pepck	TGACAGACTCGCCCTATGTG	CCCAGTTGTTGACCAAAGGC
Shp	TCTGCAGGTCGTCCGACTATTC	AGGCAGTGGCTGTGAGATGC
β-klotho	TGTCAACGGCACAGACAGAT	CGCATTCACTGCTGCTACTG

*, also known as *Rplp0*

ESM Table 4. Statistical significance of differences in the percentage of each bile acid species in *db/db* mice plasma, liver and cecum content related to ESM Fig. 2a

	(%of total BAs)	db	db AGI	p value (db vs db AGI)
Plasma	CA	1.96±0.61	28.06±7.73	0.015
	CDCA	1.45±0.38	1.89±0.47	0.328
	αMCA	0.05±0.03	0.31±0.09	0.015
	βMCA	1.15±0.51	1.61±0.59	0.382
	UDCA	0.09±0.06	0.22±0.1	0.382
	TCA	49.23±4.5	51.35±8.84	1
	TCDCA	1.41±0.31	0.7±0.21	0.038
	TαMCA	2.14±0.44	1.42±0.21	0.234
	TβMCA	6.22±0.99	1.03±0.2	<0.001
	DCA	27.04±7.03	9.67±1.75	0.05
	ωMCA	4.5±1.11	0.7±0.25	0.003
	TDCA	1.01±0.32	1.5±0.67	1
Liver	CA	0.19±0.09	1.36±0.78	0.808
	CDCA	0.04±0.01	0.02±0.01	0.461
	αMCA	0.05±0.01	0.05±0.02	0.808
	βMCA	0.58±0.15	0.14±0.06	0.016
	TCA	81.95±0.93	93.01±1.22	0.004
	TCDCA	1.82±0.28	1.17±0.28	0.368
	TαMCA	2.14±0.24	1.78±0.27	0.461
	TβMCA	10.04±1.15	1.11±0.19	0.004
	TUDCA	1.7±0.16	0.19±0.03	0.004
	DCA	0.01±0	0.03±0.03	0.461
	ωMCA	0.33±0.12	0.03±0.02	0.008
	TDCA	1.02±0.39	0.99±0.91	0.57
Cecum	CA	19.6±6.57	13.08±4.63	0.57
	CDCA	0.7±0.19	1.8±0.59	0.214
	αMCA	7.23±2.98	2.76±0.89	0.683
	βMCA	14.64±3.39	28.05±11.05	0.368
	UDCA	0.95±0.3	0.85±0.32	0.933
	TCA	11.19±5.29	10.22±8.07	1
	TαMCA	2.3±1.11	11.74±4.93	0.073
	TβMCA	2.35±1.05	11.35±2.82	0.008
	DCA	31.75±11.93	14.15±13.71	0.214
	LCA	1.47±0.64	1.31±1.07	1
	TDCA	0.52±0.21	ND	-

Date are presented as mean±SEM. ND, not detected. P values calculated by Mann-Whitney U test. CA, cholic acid; CDCA, chenodeoxycholic acid; MCA, murine cholic acid; UDCA, ursodeoxycholic acid; TCA, taurine conjugated CA; TCDCA, taurine conjugated CA; TαMCA, taurine conjugated alpha MCA; TβMCA, taurine conjugated beta MCA; TUDCA, taurine conjugated UDCA; DCA, deoxycholic acid; LCA, lithocholic acid; TDCA, taurine conjugated DCA; TLCA, taurine conjugated LCA; TωMCA, taurine conjugated omega MCA.

ESM Table 5. Statistical significance of differences in the percentage of each bile acid species in HFHS mice plasma, liver, cecum content and ileum related to Fig. 3a

(%of total BAs)	WT	WA	KO	KA	p value (WT vs WA)	p value (KO vs KA)	p value (WT vs KO)	p value (WA vs KA)
				Plasma				
CA	6.98±1.36	30.77±2.41	9.53±1.19	13.57±4.01	0.001	0.606	0.606	0.005
CDCA	1.35±0.21	3.03±0.63	0.47±0.11	0.2±0.05	0.035	0.031	0.002	0.001
αMCA	0.85±0.11	4.24±0.78	0.46±0.11	0.68±0.26	0.001	0.758	0.042	0.001
βMCA	6.91±0.83	25.24±2.07	3.95±0.69	7.34±1.88	0.001	0.21	0.012	0.001
UDCA	0.73±0.11	1.23±0.32	0.36±0.1	0.08±0.03	0.234	0.005	0.031	0.001
TCA	11.63±1.33	8.99±3.59	28.99±4.06	52.44±10.19	0.366	0.071	<0.001	0.005
TCDCA	1.52±0.42	0.64±0.31	0.78±0.18	0.14±0.04	0.181	<0.001	0.091	0.366
TαMCA	5.38±0.84	2.25±0.75	2.71±0.47	0.7±0.15	0.035	<0.001	0.023	0.101
TβMCA	11.3±1.43	4.72±1.52	7.51±0.99	9.46±3.24	0.014	0.918	0.055	0.628
TUDCA	4.57±1.28	0.71±0.23	1.73±0.39	0.4±0.13	0.005	0.005	0.042	0.181
DCA	11.41±0.98	1.15±0.97	15.2±2.49	3.55±2.31	0.001	0.008	0.47	0.534
ωMCA	24.46±4.59	15.87±5.98	13.93±2.41	7.7±3.92	0.181	0.142	0.071	0.181
TDCA	3.93±0.63	0.05±0.05	6.7±0.55	0.49±0.32	0.001	<0.001	0.008	0.628
TωMCA	6.5±0.77	0.67±0.22	6.1±1.27	2.09±0.93	0.001	0.023	0.681	0.534
				Liver				
CA	0.87±0.3	10.17±4.22	2.38±0.9	5.62±1.84	0.002	0.219	0.107	0.487
CDCA	0.08±0.02	0.05±0.01	0.04±0.01	0.03±0.02	0.173	0.006	0.107	0.011
αMCA	0.29±0.05	0.78±0.29	0.38±0.09	0.2±0.09	0.918	0.039	1	0.032
βMCA	1.06±0.13	1.65±0.64	2.2±0.5	0.77±0.27	0.468	0.014	0.203	0.525
UDCA	0.04±0.01	0.06±0.03	0.06±0.01	0.02±0.01	0.197	<0.001	0.821	0.169
TCA	41.33±1.76	55.69±4.14	61.93±2.7	81.97±3.84	0.016	<0.001	<0.001	<0.001
TCDCA	2.13±0.25	2.01±0.27	0.88±0.08	0.39±0.08	0.863	<0.001	<0.001	<0.001
TαMCA	7.41±0.6	4.93±1.02	3.52±0.34	0.87±0.3	0.016	<0.001	<0.001	<0.001
TβMCA	37.26±2.3	23.08±3.34	18.97±1.97	6.77±1.49	0.003	<0.001	<0.001	<0.001
TUDCA	2.44±0.14	1.19±0.25	1.23±0.11	0.26±0.06	0.002	<0.001	<0.001	<0.001

DCA	0.43±0.2	0.11±0.05	0.22±0.09	0.21±0.09	0.043	0.347	0.381	0.88
ωMCA	1.62±0.36	0.24±0.16	1.64±0.31	0.21±0.08	<0.001	<0.001	0.872	0.151
TDCA	4.63±0.6	0.02±0.02	6.37±1.23	2±1.02	<0.001	0.004	0.418	0.004
TLCA	0.19±0.05	0.01±0	0.08±0.02	0.02±0.01	<0.001	<0.001	0.05	1
TωMCA	0.22±0.03	0.01±0	0.09±0.01	0.04±0.01	<0.001	0.008	0.001	0.651
Cecum content								
CA	3.49±2.45	24.43±4.23	5.5±2.14	17.83±8	0.002	0.268	0.234	0.429
CDCA	0.16±0.09	0.91±0.15	0.11±0.04	ND	0.002	0.048	1	0.004
αMCA	5.03±1.85	10.78±1.69	4.78±0.48	1.56±0.31	0.041	0.003	0.051	0.004
βMCA	8.68±2.68	39.68±3.93	8.06±0.83	18.48±7.55	0.002	0.639	0.445	0.052
UDCA	ND	0.94±0.13	0.01±0.01	ND	-	-	-	-
TCA	0.43±0.12	ND	0.18±0.06	0.72±0.38	-	0.343	0.035	-
TαMCA	0.11±0.02	0.72±0.3	0.08±0.02	ND	0.065	0.003	0.295	0.017
TβMCA	0.57±0.14	9.13±4.98	0.22±0.05	ND	0.009	0.003	0.035	0.004
DCA	20.82±3.34	0.1±0.01	22.56±1.89	35.85±18.23	0.002	0.639	1	0.004
LCA	2.2±0.4	ND	0.92±0.17	0.57±0.36	-	0.043	0.051	-
ωMCA	55.7±5.1	12.93±3.58	53.85±2.55	23.03±8.15	0.002	0.03	0.534	0.429
TDCA	0.13±0.03	ND	0.45±0.16	0.05±0.03	0.002	0.003	0.051	0.329
Ileum								
CA	11.09±5.46	32.39±4.14	24.6±4.07	55.75±8.19	0.022	0.01	0.04	0.059
CDCA	0.31±0.15	0.75±0.19	0.13±0.03	0.12±0.03	0.022	0.798	0.281	0.001
αMCA	1.12±0.59	6.67±2.49	0.76±0.16	0.61±0.2	0.051	0.574	0.694	0.02
βMCA	2.13±0.84	12.74±4.2	1.74±0.38	4.36±1.6	0.035	0.574	1	0.081
UDCA	0.13±0.05	0.57±0.17	0.08±0.02	0.04±0.01	0.051	0.13	0.694	0.001
TCA	29.24±4.79	13.39±8.43	38.78±5.29	25.85±8.66	0.073	0.234	0.232	0.414
TCDCA	1.4±0.34	0.71±0.12	0.35±0.05	0.16±0.07	0.234	0.038	<0.001	0.001
TαMCA	7.56±1.14	4.22±0.53	4.19±0.62	2.02±1.17	0.073	0.05	0.029	0.142
TβMCA	37.68±5.33	23.05±7.74	19.71±2.36	5.31±1.57	0.101	<0.001	0.021	0.029
TUDCA	1.56±0.37	0.86±0.23	0.51±0.09	0.3±0.1	0.234	0.105	0.054	0.043
DCA	0.92±0.54	0.06±0.02	1.17±0.23	1.15±0.56	0.001	0.328	0.094	0.029
ωMCA	4.89±1.66	4.5±1.92	4.98±1.02	3.84±1.6	0.366	0.279	0.867	1

TDCA	1.66±0.48	0.06±0.03	2.83±0.93	0.41±0.31	0.001	0.005	0.152	0.142
TLCA	0.06±0.01	ND	0.03±0	0.01±0	-	<0.001	0.072	-
TωMCA	0.25±0.06	0.04±0.02	0.13±0.02	0.06±0.06	0.008	0.01	0.232	0.181

Date are presented as mean±SEM. ND, not detected. P values calculated by Mann-Whitney U test

ESM Table 6. Metabolic improvements in human type 2 diabetes pre and post AGI treatment

	Pre AGI treatment	Post AGI treatment	*p* value
Age (years)	53.32±8.49	/	/
Sex (male)	7	/	/
Height (cm)	171.10±7.22	/	/
BW (kg)	77.19±10.35	73.89±10.73	0.0217
BMI (kg/m^2)	26.34±3.03	25.22±3.37	0.0161
G0 (mmol/l)	7.97±1.33	6.35±0.74	0.005
G30 (mmol/l)	10.58±1.80	7.65±1.74	0.0012
G60 (mmol/l)	14.40±2.52	9.84±1.96	0.0004
G120 (mmol/l)	14.66±2.75	9.65±1.75	0.0002
G180 (mmol/l)	11.88±3.64	7.66±2.70	0.0138
INS0 (pmol/l)	82.33±46.11	59.79±36.69	0.0097
INS30 (pmol/l)	168.45±115.56	129.75±96.88	0.274
INS60 (pmol/l)	347.14±207.41	242.49±158.40	0.1845
INS120 (pmol/l)	458.81±279.92	283.20±141.40	0.0265
INS180 (pmol/l)	337.97±234.06	212.37±167.38	0.0017
CP0 (nmol/l)	1.01±0.28	0.83±0.24	0.0493
CP30 (nmol/l)	1.38±0.47	1.10±0.36	0.0606
CP60 (nmol/l)	2.07±0.74	1.61±0.54	0.0631
CP120 (nmol/l)	3.03±0.91	2.34±0.47	0.0129
CP180(nmol/l)	2.78±1.02	2.06±0.73	0.0041
HbA1C (%)	7.95±0.92	6.20±0.43	0.0004
HbA1C (mmol/mol)	49.29±5.704	38.44±2.66	0.0004
HOMAIR (pmol×mmol)	28.65±18.81	17.47±11.98	0.0083

Paired t test，$p<0.05$ considered to be statistical significance

BW: body weight, BMI: body mass index, G0-G180: blood glucose levels at 0, 30, 60, 120, 180 min during oral glucose tolerance, INS0-180, serum insulin levels at 0, 30, 60, 120, 180 min during oral glucose tolerance, CP0-CP180, serum C-peptide at 0, 30, 60, 120, 180 min during oral glucose tolerance, HbA1C, haemoglobin A1C, HOMAIR, homeostasis model assessment index for insulin resistance.

ESM Table 7. Two step hyperglycaemia clamp

		pre-week 0	post-week 12	*p*
**Step 1**				
**Ave BG mmol/l**		14.6±0.2	14.3±0.2	0.006
**GIR (mmol/l per kg per min)**		6.0±0.5	7.9±0.8	0.004
**AUCins (pmol/l per min)**	**Phase I**	1039.2±123.3	1051.7±163.0	0.945
	**Phase II**	8389.3±868.5	9378.4±2083.2	0.583
**Step 2**				
**Ave BG mmol/l**		15.1±0.1	14.0±0.2	<0.001
**GIR (mmol/l per kg per min)**		4.5±0.5	11.7±0.8	<0.001
**AUCins (pmol/l per min)**	**Phase I**	3266.5±514.8	4640.3±970.1	0.077
	**Phase II**	17871.0±2795.8	23421.1±4367.8	0.254

12 weeks of AGI treatment significantly decreased GIR of the clamp and showed a trend of improved acute insulin secretion (Phase I) in the Step 2. Ave BG： average blood glucose level clamped during the experiment. GIR: glucose infusion rate; AUCins: area under curve for multiple measurements of plasma insulin level. Data were presented as mean ± S.D., Student t test.

ESM Table 8. Islet lipotoxicity related genes

Symbol	GeneID	LnRatio(WA/WT)	q-value	Up/Down	References
Cat	12359	0.134842081	0.0189369	-	Kace J. Prentice et al. Cell Metabolism. (2014) [11]
Cpe	12876	0.049131061	2.70E-74	-	
Ucp2	22228	-0.246916569	1.17E-197	-	
Acadvl	11370	-0.144090208	1.13E-12	-	Tianyi Tang et al. Cell Metabolism. (2013) [12]
Cpt1b	12895	0.278957081	0.0042817	-	
Cpt2	12896	0.028745675	0.0723977	-	
Hadhb	231086	0.007239168	0.2270785	-	
Mdh2	17448	0.056078276	0.0001881	-	
Pdk4	27273	0.28485563	3.32E-16	-	
Pnpla2	66853	0.181126939	3.49E-17	-	
Sdhb	67680	-0.030436229	0.0943754	-	
Abcc8	20927	0.091641918	2.78E-127	-	Marc Prentki et al. Cell Metabolism. (2013) [13]
Abhd6	66082	-0.075559508	0.0139342	-	
Cpt1a	12894	-0.045296797	0.0024636	-	
Cpt1c	78070	0.123585756	0.0029551	-	
Idh1	15926	0.146786014	3.95E-06	-	

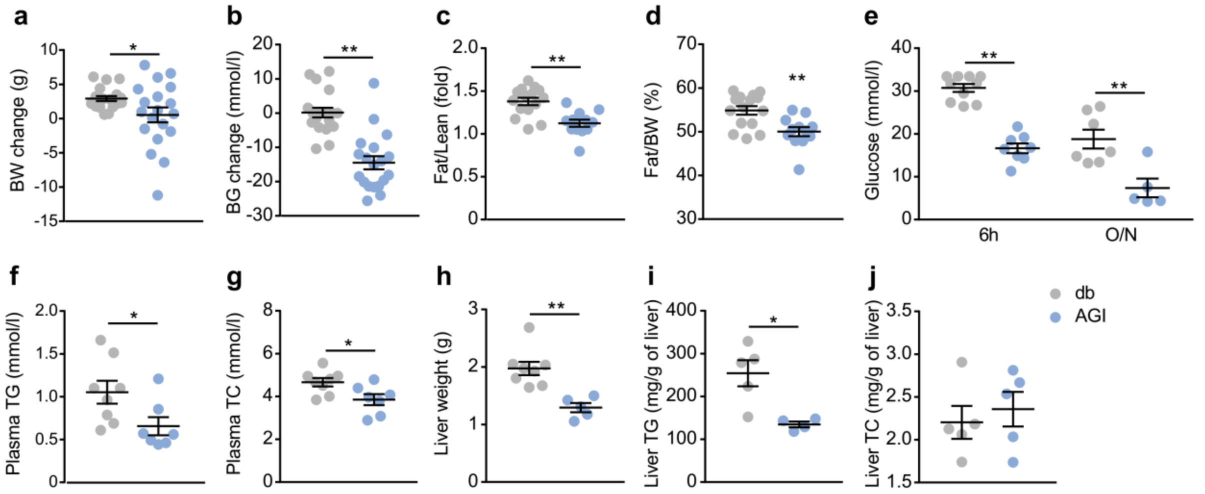

ESM Figure 1. Other metabolic phenotypes in *db/db* mice after AGI treatment. (a) AGI reduced total body weight (BW) after 24 days treatment. n=19-24 each group. (b) AGI reduced blood glucose (BG) levels after 24 days treatment. n=19-24 each group. (c) AGI reduced ratio of fat vs lean mass, n=12-15 each group. (d) AGI reduced body fat composition, n=12-15 each group. (e) AGI reduced fasting blood glucose (FBG) after treatment, 6 hours (6h) fasting (left) and overnight (O/N) fasting (right), n=5-12 each group. AGI reduced blood lipids of mice after treatment: triglycerides (TG) (f) and total cholesterol (TC) (g), n=7-11 each group. (h) AGI reduced Liver weight, n=5-8 each group. AGI reduced liver content of TG (i) and TC (j), n=4-8 each group. Grey circles, db group (Veh treated); blue circles, AGI group (AGI treated). Data are presented as mean ± SEM. * p <0.05, ** p <0.01, two tailed Student's t test.

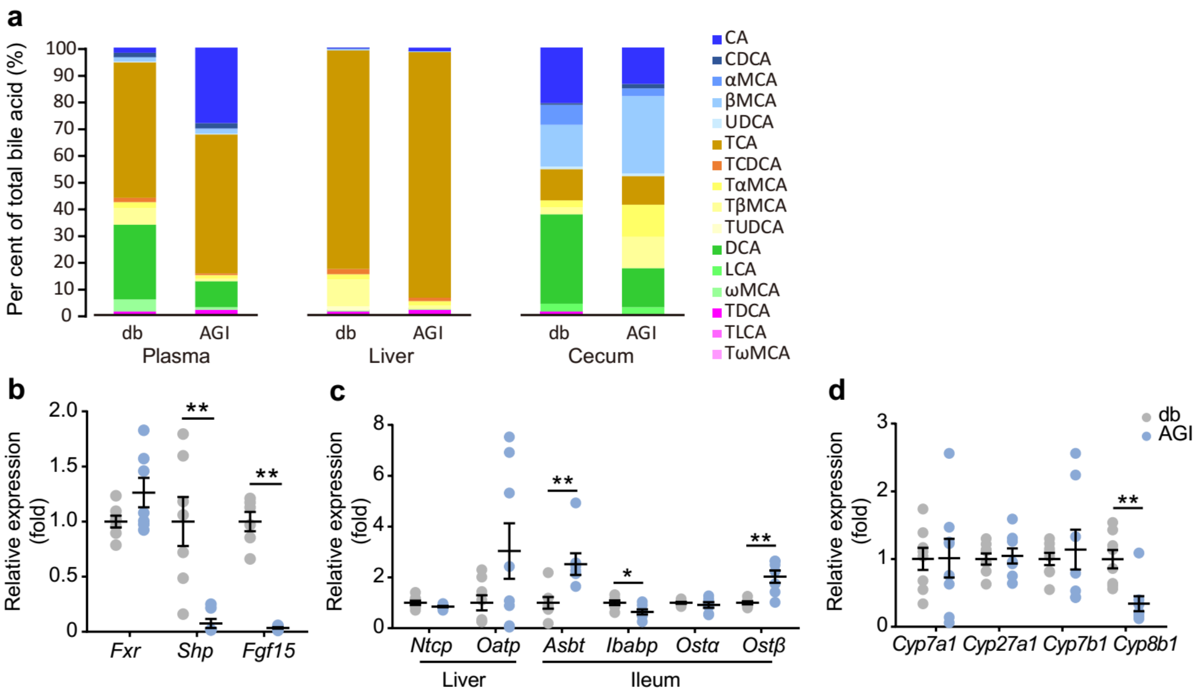

ESM Figure 2. AGI changed enterohepatic BA component and BA signalling in *db/db* mice. (a) Stacked bar plot of BA pools in plasma, liver and cecum content, n=7-9 each group. (b-d) Changes of mRNA levels of genes regulating ileum FXR signalling (b), BA transportation (c) and liver BA synthesis (d), n=7 each group. Grey circles, db group (Veh treated); blue circles, AGI group (AGI treated). Data are presented as mean ± SEM. ** p <0.01, two tailed Student's t test. CA, cholic acid; CDCA, chenodeoxycholic acid; MCA, murine cholic acid; UDCA, ursodeoxycholic acid; TCA, taurine conjugated CA; TCDCA, taurine conjugated CA; TαMCA, taurine conjugated alpha MCA; TβMCA, taurine conjugated beta MCA; TUDCA, taurine conjugated UDCA; DCA, deoxycholic acid; LCA, lithocholic acid; TDCA, taurine conjugated DCA; TLCA, taurine conjugated LCA; TωMCA, taurine conjugated omega MCA.

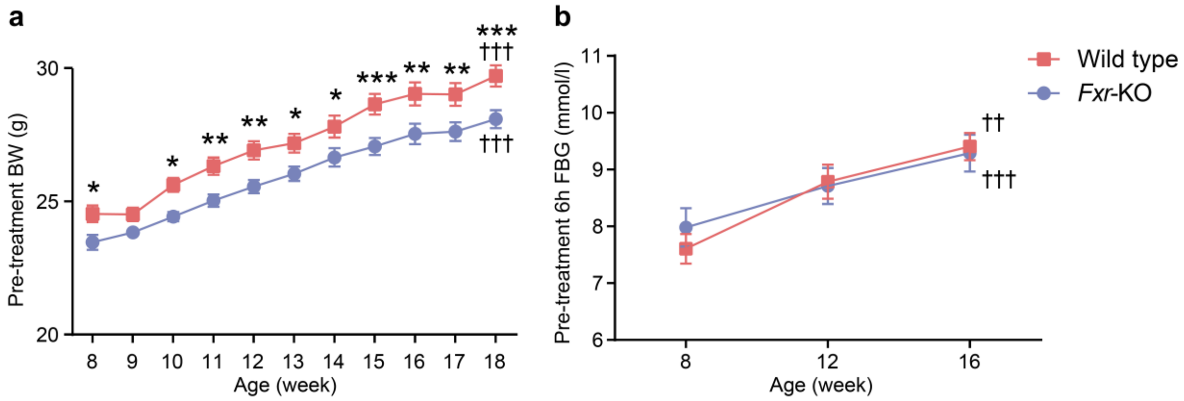

ESM Figure 3. Metabolic phenotype of Wild type and *Fxr*-KO mice after HFHS diet before AGI treatment. (a) Body weight (BW) every week on HFHS diet. n=20-21 each group, (b) Fasting blood glucose (FBG) values detected every 4 weeks on HFHS diet. Blue circles: *Fxr*-KO, *Fxr* knockout mice; red square: wild type. n=20-21 each group. * p <0.05, ** p <0.01, ***p <0.001, wild type vs *Fxr*-KO mice at each time point of measurement, †† p <0.01, ††† p <0.001, vs baseline value in each genotype, two-way ANOVA. Data are presented as mean ± SEM.

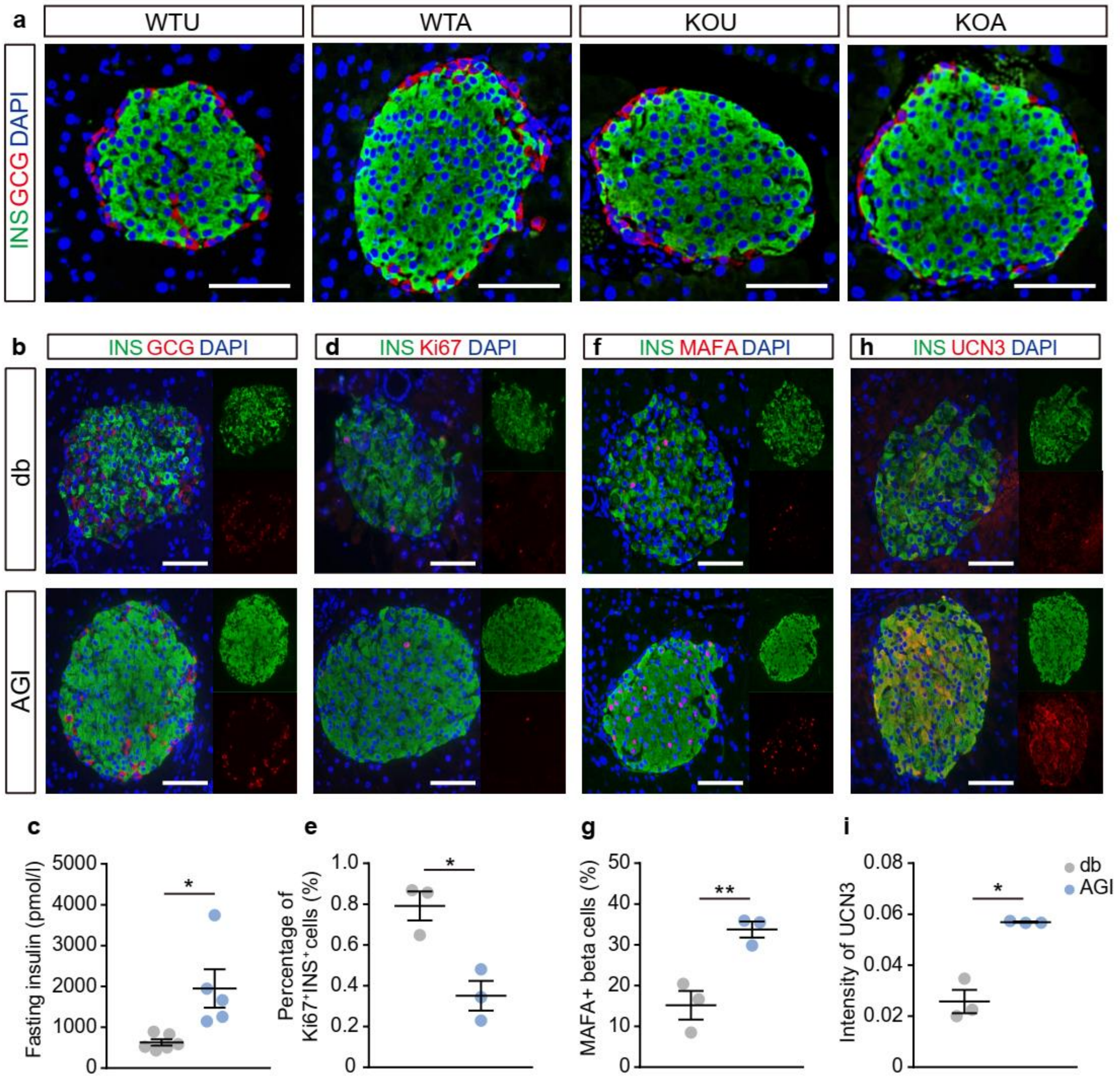

ESM Figure 4. The morphology and function changes of endocrine pancreas after AGI treatment. (a) Islet structure, representative image of pancreas insulin/glucagon immunolabeling of HFHS fed mice. n=3 each group. Scale bars, 100 μm. (b) Islet structure, representative image of pancreas insulin/glucagon immunolabeling of *db/db* mice, n=3 each group. Scale bars, 100 μm. (c) Fasting plasma insulin levels after treating AGI, n=5 each group. (d-e) Representative figures of islet Ki67 immunolabelling (d) and comparison of Ki67 positive beta cell percentage (e), n=3 each group. Scale bars, 100 μm. (f-g) Representative figures of islet MafA immunolabeling (f) and comparison of MafA positive beta cell percentage (g), n=3 each group. Scale bars, 100 μm. (h-i) Representative figures of islet UCN3 immunolabeling (h) and calculation of staining intensity (i) in beta cells. n=3 each group. Scale bars, 100 μm. MafA, v-maf musculoaponeurotic fibrosarcoma oncogene family, protein A; UCN3, Urocortin 3. Data are presented as mean ± SEM. * p <0.05, ** p <0.01, db vs AGI, two tailed Student's t-test. Grey circles, db group (Veh treated); blue circles, AGI group (AGI treated).

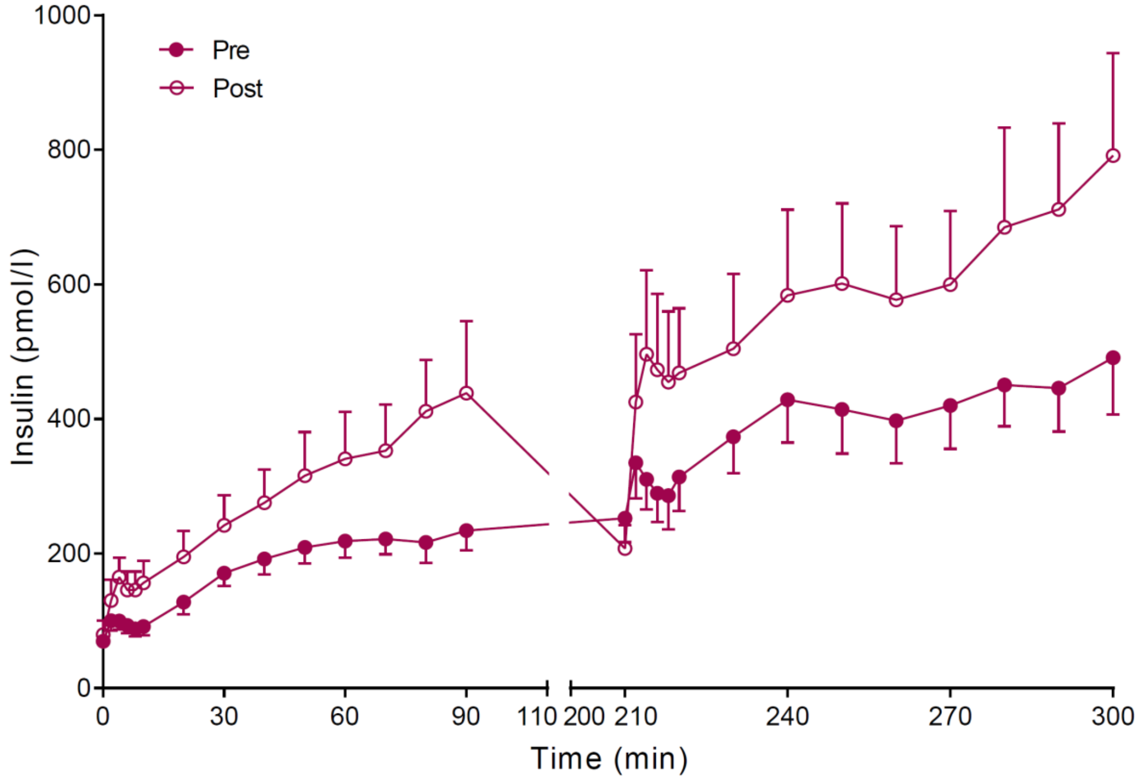

ESM Figure 5. Two-step hyperglycaemic clamp in human type 2 diabetes treated with AGI. Plot of insulin secretion responding to hyperglycaemia in type 2 diabetes patients before and after 3 months AGI treatment. Insulin levels were measured during the clamp experiment at multiple time points. Full red circles, Pre: pre-treatment levels, n=10; Empty red circles, Post: post treatment levels, n=9. Data are presented as mean ± SEM. Multiple t-test.

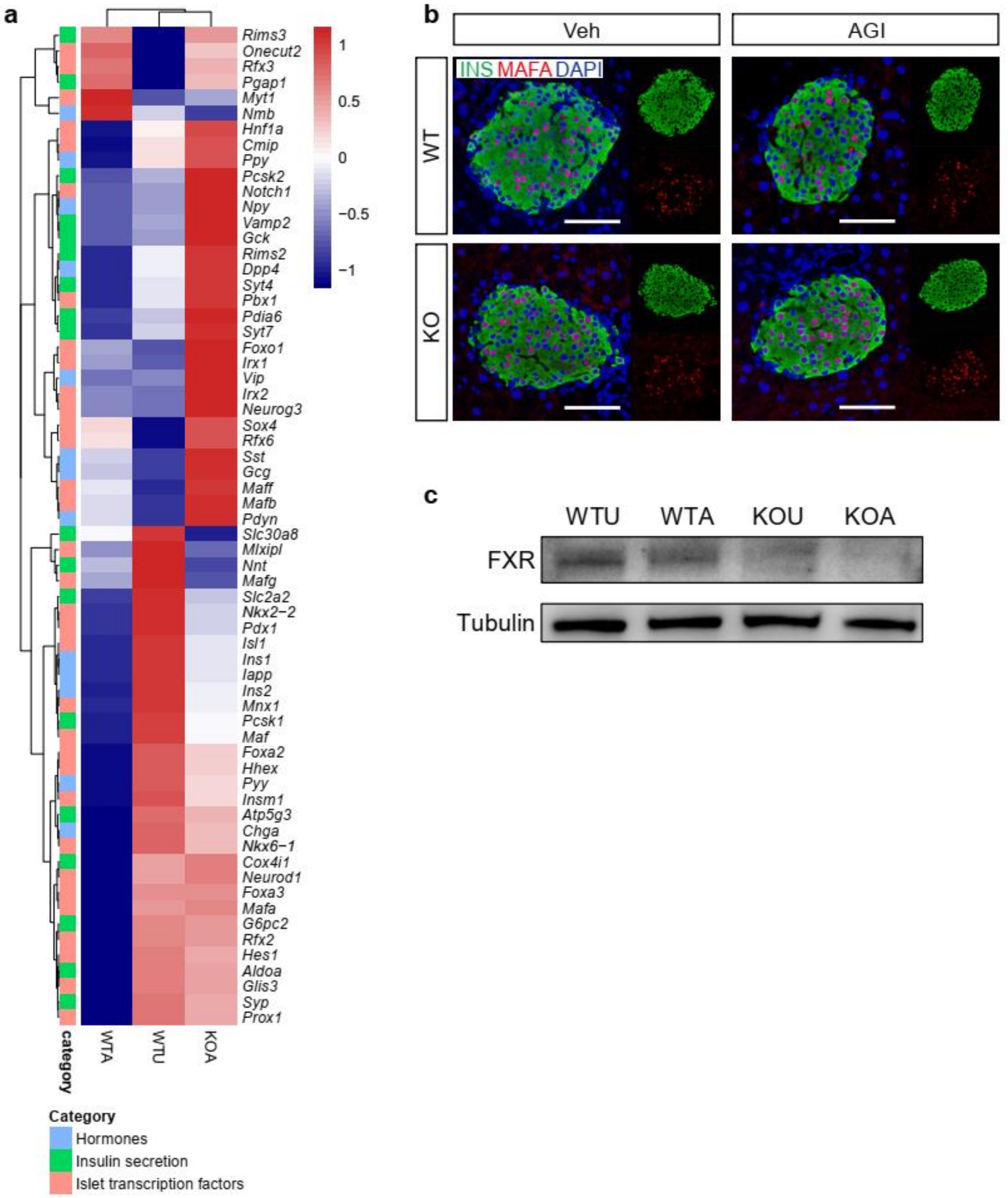

ESM Figure 6. RNA sequencing data revealed changes of key islet TF expressions after AGI treatment. (a) Heatmap of key islet TF expressions, n=3 each group, z-score of normalized relative expression of genes in row. (b) Representative image of immunolabeling of MafA and insulin in pancreatic islets of HFHS fed mice, n=3 each group. Scale bars, 100 μm. (c) Western blot of FXR protein in islets of HFHS fed mice, n=3 each group.

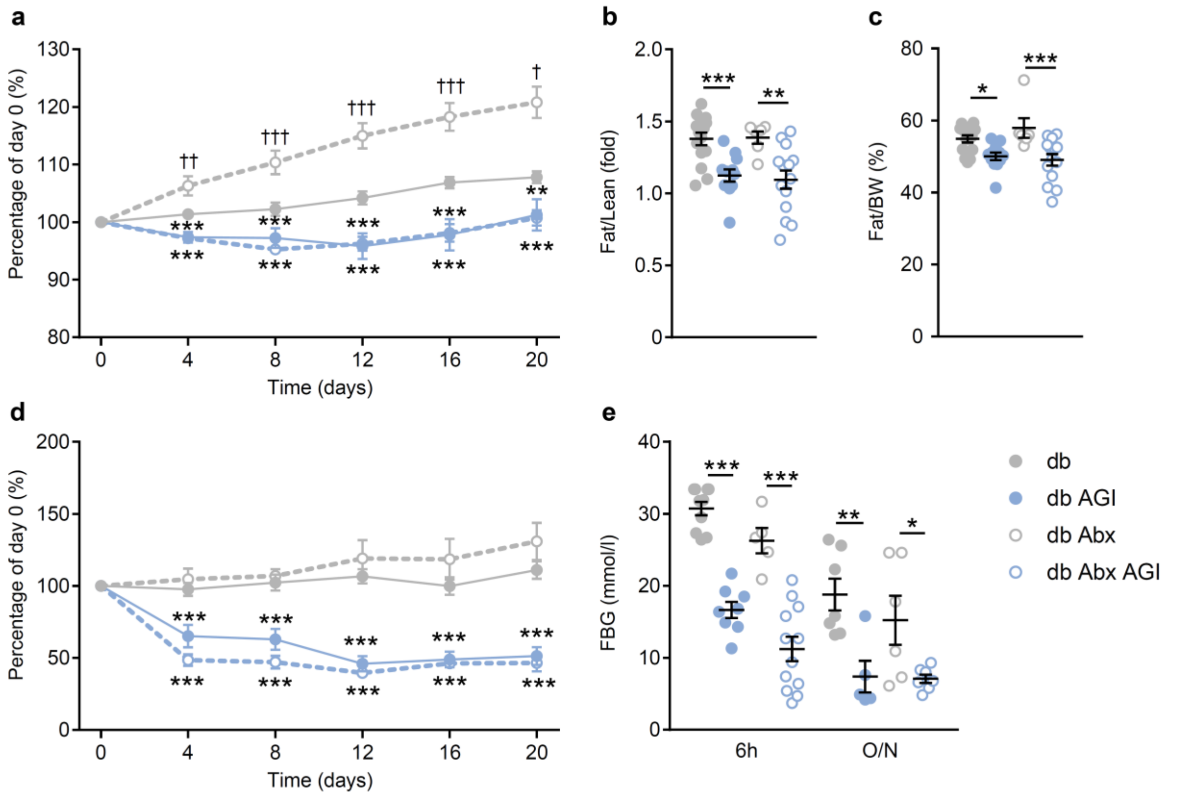

ESM Figure 7. Metabolic phenotypes of *db/db* mice after Abx and AGI treatment. (a) Change of body weight (BW) of baseline (db: 38.4±1.0 g, db AGI: 39.1±0.9 g, db Abx: 34.5±0.7 g, db Abx AGI: 37.7±1.1 g, mean± SEM) every 4 days during AGI treatment. (b) Ratio of fat vs lean mass, (c) Percentage of body fat mass in the end of AGI treatment. (d) Change of fed-state BG from baseline (db: 28.0±1.1 mmol/l, db AGI: 29.9±0.8 mmol/l, db Abx: 24.8±1.4 mmol/l, db Abx AGI: 25.2±0.9 mmol/l, mean± SEM) every 4 days during AGI treatment. (e) Fasting blood glucose (FBG) in the end of AGI treatment, n=6-25 each group. Data are presented as mean ± SEM. * p <0.05, ** p <0.01, *** p <0.001, db vs db AGI or db Abx vs db Abx AGI; † p <0.05, †† p <0.01, ††† p <0.001 db vs db Abx, two-way ANOVA. Grey full circles: db group, untreated control *db/db* mice; blue full circles: db AGI group, AGI treated *db/db* mice; grey empty circles: db Abx group, untreated control *db/db* mice with pretreatment of antibiotics; blue empty circles: db Abx AGI group, AGI treated *db/db* mice with pretreatment of antibiotics.

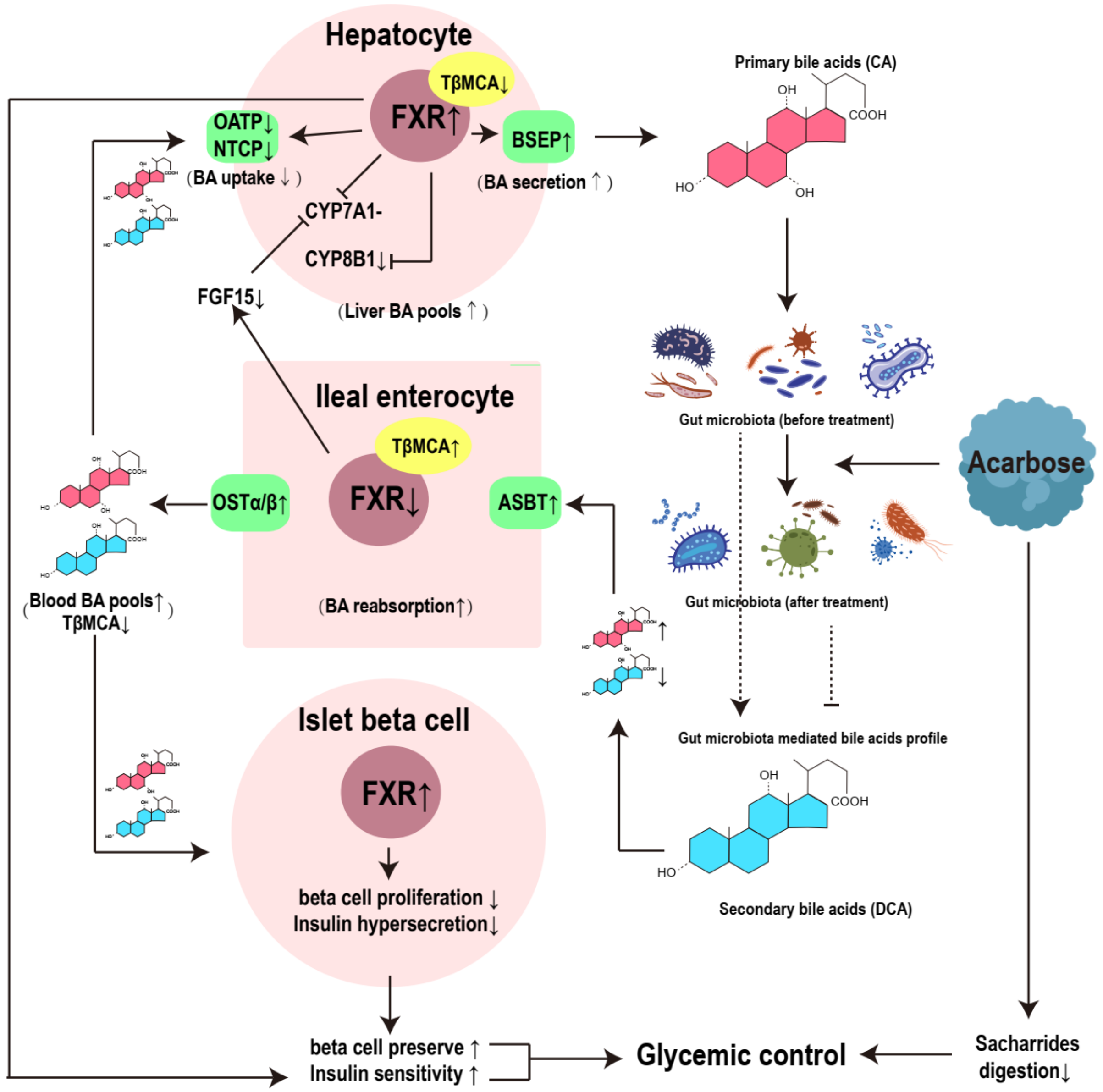

ESM Figure 8. Schematic map for mechanism underlying AGI regulating BA pool and signalling to improve its therapeutic effect.

ESM References

[1] Fu L, Qiu Y, Shen L, et al. (2018) The delayed effects of antibiotics in type 2 diabetes, friend or foe? The Journal of endocrinology 238(2): 137-149. 10.1530/joe-17-0709

[2] Magoc T, Salzberg SL (2011) FLASH: fast length adjustment of short reads to improve genome assemblies. Bioinformatics (Oxford, England) 27(21): 2957-2963. 10.1093/bioinformatics/btr507

[3] Amann RI, Binder BJ, Olson RJ, Chisholm SW, Devereux R, Stahl DA (1990) Combination of 16S rRNA-targeted oligonucleotide probes with flow cytometry for analyzing mixed microbial populations. Applied and environmental microbiology 56(6): 1919-1925

[4] Suez J, Zmora N, Zilberman-Schapira G, et al. (2018) Post-Antibiotic Gut Mucosal Microbiome Reconstitution Is Impaired by Probiotics and Improved by Autologous FMT. Cell 174(6): 1406-1423.e1416. 10.1016/j.cell.2018.08.047

[5] Gu Y, Lindner J, Kumar A, Yuan W, Magnuson MA (2011) Rictor/mTORC2 is essential for maintaining a balance between beta-cell proliferation and cell size. Diabetes 60(3): 827-837. 10.2337/db10-1194

[6] Chen K, Liu J, Liu S, et al. (2017) Methyltransferase SETD2-Mediated Methylation of STAT1 Is Critical for Interferon Antiviral Activity. Cell 170(3): 492-506.e414. 10.1016/j.cell.2017.06.042

[7] Wang L, Feng Z, Wang X, Wang X, Zhang X (2010) DEGseq: an R package for identifying differentially expressed genes from RNA-seq data. Bioinformatics (Oxford, England) 26(1): 136-138. 10.1093/bioinformatics/btp612

[8] Storey JD, Tibshirani R (2003) Statistical significance for genomewide studies. Proc Natl Acad Sci U S A 100(16): 9440-9445. 10.1073/pnas.1530509100

[9] Zhang Y, Chi J, Wang W, et al. (2015) Different effects of two dipeptidyl peptidase-4 inhibitors and glimepiride on beta-cell function in a newly designed two-step hyperglycemic clamp. Journal of diabetes 7(2): 213-221. 10.1111/1753-0407.12175

[10] Gu Y, Wang X, Li J, et al. (2017) Analyses of gut microbiota and plasma bile acids enable stratification of patients for antidiabetic treatment. Nature communications 8(1): 1785. 10.1038/s41467-017-01682-2

[11] Prentice KJ, Luu L, Allister EM, et al. (2014) The furan fatty acid metabolite CMPF is elevated in diabetes and induces beta cell dysfunction. Cell metabolism 19(4): 653-666. 10.1016/j.cmet.2014.03.008

[12] Tang T, Abbott MJ, Ahmadian M, Lopes AB, Wang Y, Sul HS (2013) Desnutrin/ATGL activates PPARdelta to promote mitochondrial function for insulin secretion in islet beta cells. Cell metabolism 18(6): 883-895. 10.1016/j.cmet.2013.10.012

[13] Prentki M, Matschinsky FM, Madiraju SR (2013) Metabolic signaling in fuel-induced insulin secretion. Cell metabolism 18(2): 162-185. 10.1016/j.cmet.2013.05.018
